# Reverse electron transfer at mitochondrial complex I restrains dopaminergic neuron activity to promote early-life sleep in *Drosophila*

**DOI:** 10.64898/2026.02.22.707308

**Published:** 2026-02-23

**Authors:** Jeffrey B. Rosa, Hayle H. Kim, Jenny Luong, Jianing Yang, Peyton Yee, Allen Yan, Anyara Rodriguez, Matthew S. Kayser

**Affiliations:** 1 Department of Psychiatry, Perelman School of Medicine, University of Pennsylvania, Philadelphia, PA, USA; 2 Chronobiology and Sleep Institute, Perelman School of Medicine, University of Pennsylvania, Philadelphia, PA, USA; 3 College of Arts and Sciences, University of Pennsylvania, Philadelphia, PA, USA; 4 Department of Neuroscience, Perelman School of Medicine, University of Pennsylvania, Philadelphia, PA, USA

## Abstract

Sleep architecture and depth undergo profound changes across early life. In many species, including *Drosophila melanogaster*, juvenile animals exhibit elevated sleep drive and deeper sleep states relative to adults, a process linked to reduced activity of wake-promoting dopaminergic neurons (DANs). To identify cell-intrinsic mechanisms regulating developmental sleep, we profiled gene expression in juvenile and mature DANs and performed a targeted RNAi screen of genes with higher juvenile expression. From this screen, we found that the magnitude of mitochondrial complex I (MCI) disruption produced distinct behavioral outcomes. Severe MCI loss-of-function caused locomotor deficits due to mitochondrial dysfunction and reduced neuronal activity. Surprisingly, partial MCI inhibition preserved mitochondrial integrity but resulted in sleep loss, with a most pronounced impact on juvenile adult sleep fragmentation and depth. We demonstrate that dopaminergic neuron activity in juvenile flies is sensitive to the Coenzyme Q redox state with a low CoQ/CoQH2 promoting sleep depth by restraining DAN activity. Our results are consistent with a model in which the reverse transfer of electrons from CoQH2 to NAD+ at MCI limits DAN activity. By dissociating changes in CoQ redox state from catastrophic mitochondrial failure, this work indicates that sleep phenotypes may serve as sensitive indicators of emerging mitochondrial dysfunction, with implications for understanding the developmental origins of neurodegenerative vulnerability.

## Introduction

Sleep characteristics change across the lifespan. Across the animal kingdom, juvenile animals experience longer and deeper sleep states than their adult counterparts. In humans, newborns spend up to 70% of the day asleep, and, compared to adults, newborn sleep is disproportionately enriched in rapid eye movement (REM) sleep(1, 2)^,^. Non-REM sleep in newborns is also defined by unique patterns of cortical brain activity leading to a differences in the distribution and shape of sleep spindles, which are associated with memory consolidation. At a molecular level, human genome wide association studies in adolescents and adults point to unique genetic variants impacting sleep quality at these different ages(3). Likewise, genetic regulators of sleep appear to be at least partially distinct in early life compared to maturity in animal models(4, 5). These findings suggest that early-life sleep is under distinct genetic and neural control, resulting in its unique attributes. Yet juvenile sleep does not persist indefinitely: instead, sleep architecture and depth undergo a coordinated maturation across early life. The molecular and circuit-level mechanisms that actively drive this transition from juvenile to adult sleep are unknown.

The ontogenetic hypothesis of sleep postulates an essential role for juvenile sleep in brain and behavior maturation. Early-life sleep disruption in both invertebrates and vertebrates causes later deficits in social behavior associated with synaptic abnormalities in the brain(4–7). These observations indicate that early-life sleep could play instructive and/or permissive roles during typical brain development. Moreover, early-life sleep disturbances are a convergent comorbidity of neurodevelopmental disorders, with the severity of sleep disturbances correlating with the severity of social deficits(8, 9) This suggests that sleep disruption in children with atypical neural development may be a driver of later behavioral and cognitive deficits. Unlike hard-wired synaptic defects, early-life sleep may be sufficiently malleable to provide a potential therapeutic avenue to improve behavior development and quality of life in these children and their caregivers.

The fruit fly *Drosophila melanogaster* has been a powerful model system to investigate the genetic and circuit regulation of early-life sleep. Compared to their mature adult (~6–10 days old) counterparts, juvenile adult flies (0–1 day old) fall asleep faster, sleep more, and experience deeper sleep states(5). Previous work has found that high sleep drive in juvenile flies is facilitated by relatively hypoactive dopaminergic neurons, which normally promote arousal(5)^.^ During the first week of adult life, dopaminergic activity increases, leading to a reduction in sleep duration, in part through greater inhibition of sleep-promoting neurons. Work in mammalian models likewise implicates dopamine signaling in changes to sleep across development(4). However, the cell intrinsic processes acting in DANs to restrict their activity during excess sleep periods of juveniles and/or ramp up their synaptic output over time to mediate sleep maturation to adulthood remain unknown.

To identify cell-autonomous mechanisms controlling juvenile sleep, we carried out RNA sequencing of juvenile and mature DANs, followed by transgenic RNAi screening to identify genes required in DANs for normal juvenile sleep drive. We found that juvenile DANs showed higher expression of numerous metabolic pathway genes, most notably components of the oxidative phosphorylation (OXPHOS) pathway responsible for ATP synthesis, implicating metabolic differences between juvenile and mature DANs. Through RNAi screening, we identified multiple hits in Mitochondrial Complex I (MCI) as causing apparent opposing phenotypes, leading to either short sleep or locomotor hypoactivity. Using multiple genetic and biochemical approaches, we demonstrate that hypoactivity is caused by severe MCI deficiency leading to early DAN hypofunction, while sleep loss is explained by a partial inhibition of MCI that leaves mitochondrial function largely intact but impairs reverse electron transfer (RET) at Complex I. This work identifies a novel role for dopaminergic MCI in restraining functional DAN output to control sleep drive and raises the possibility that early life sleep is connected to future neurodegenerative processes.

## Results

### Transcriptomic analysis of juvenile and adult dopaminergic neurons.

**Figure 1:**
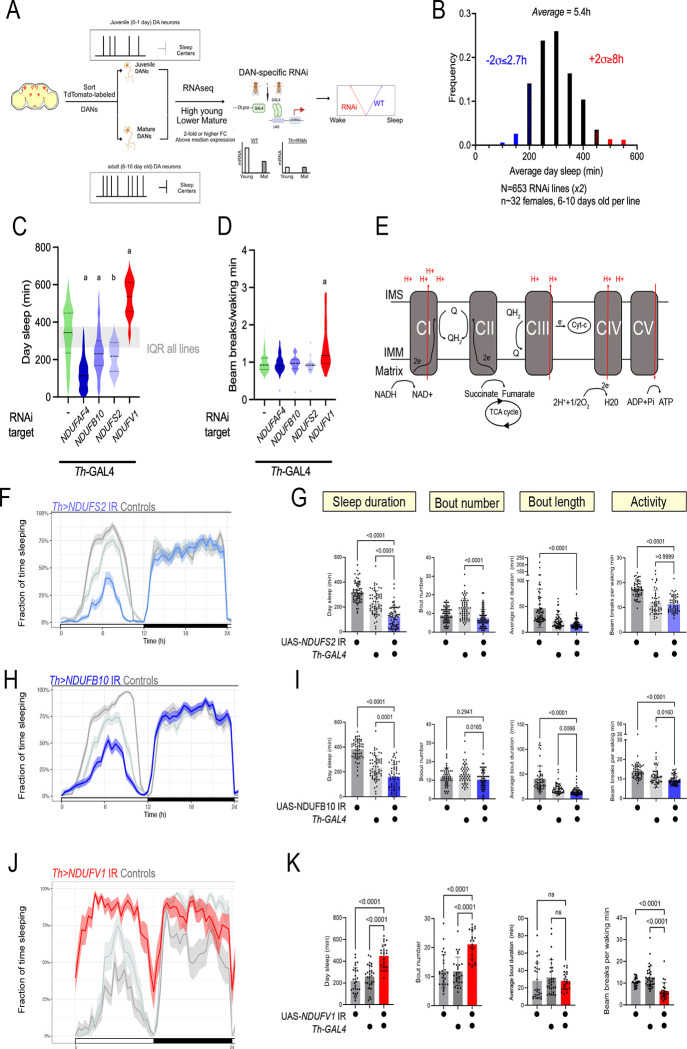
Mitochondrial Complex I loss-of-function in dopaminergic neurons causes aberrant sleep (A) Workflow of RNAi-based sleep screen using high throughput activity monitoring. (B) Average daytime (ZT0–12) sleep duration from Th>RNAi screen conducted in 6–10 day old adult flies (n~32 per genotype, 2 biological replicates). Th>RNAi genotypes with day sleep values ≥2 standard deviations from mean were selected as hits. Short-sleepers represented in blue, long-sleepers in red. (C) Knock-down of Mitochondrial Complex I (MCI) subunits caused either sleep loss (blue) or excess sleep (red). Grey shading represents the range of the middle 50% of RNAi lines tested. (a: p<0.0001 Kruskal-Wallis test with Dunn’s multiple testing comparisons; b: p=0.0032 Kruskal-Wallis with Dunn’s multiple testing comparisons; all comparisons to Th>+ control) (D) Waking activity of Th>MCI RNAi conditions as assayed using single-beam activity monitors. (a: p<0.0001 Kruskal-Wallis with Dunn’s multiple testing comparisons, compared to *Th*>+ ). (E) MCI is an entry point for reducing equivalents from glycolysis and the TCA cycle, oxidizing NADH and shuttling electrons to the mobile electron carrier Coenzyme Q, coupled to H+ translocation. (F) Sleep per 30 min windows across a 12h:12h light: dark period measured using multi-beam activity monitoring: *Th>NDUFS2* IR trace (average with 95% confidence interval shaded) is in blue and controls in grey. (G) Sleep metrics during ZT0–12 for *Th>NDUFS2* IR and controls. (H) Sleep per 30 min windows across a 12h:12h light:dark period measured using multi-beam activity monitoring: Th>*NDUFB10* IR trace is in blue and controls in grey. (I) Sleep metrics during ZT0–12 for *Th>NDUFB10* IR flies (blue) and controls (grey). (J, K) When assayed using multi-beam activity monitoring, Th>*NDUFV1* IR flies (red) exhibited increased quiescence associated with reduced locomotor activity. P values computed using either Kruskal-Wallis tests with Dunn’s multiple-testing comparisons or Welch ANOVA with Dunnett’s T3 multiple-testing comparisons.

Maturation of sleep patterns across early life involves changes to dopaminergic neuron (DAN) activity, as high juvenile sleep drive requires dopaminergic hypoactivity. We hypothesized that transcriptional differences between juvenile and mature adult DANs might play a role in this functional change. We used fluorescence activated cell to isolate DANs from brains of juvenile (0–1 day old) and mature (6–10 day old) adult flies (Figure 1A). Focusing on genes with higher expression in juvenile DANs, we observed an enrichment of GO terms for metabolic pathways, vacuolar ATPase components, redox biology and synaptic signaling (Supplemental Figure 1A). Notably, components of the oxidative phosphorylation pathway were the most significantly enriched using both GO analysis and KEGG pathway analysis (Supplemental Figure 1 A,B), including multiple regulators of the Coenzyme Q/Ubiquinone redox state. These results suggest that DANs in the juvenile adult brain have heightened demand for metabolic enzymes.

Based on the DAN transcriptional analysis, we next sought to assess the role of DEGs in sleep. To enrich for genes with potential large effects in regulating DAN activity, we narrowed our focus to genes with both i) higher juvenile expression and ii) high expression across all conditions (above-median base expression) (Supplemental Figure 1C). We then used a *Th-GAL4* driver to express dsRNAs/shRNAs against these genes in dopaminergic neurons and used high throughput activity monitoring to track sleep in 6–10 day old female flies (n~32 flies, across two biological replicates) under 12:12h light-dark conditions (Figure 1A). Mature adult, and not juvenile flies, were used to increase the throughput of the screen. We focused on changes to day sleep, since ontogenetic sleep differences are most pronounced for this period. Across all RNAi lines (n=653), flies slept on average ~5.4h during the day (Figure 1B). Using a two standard deviation (2SD) cut-off, we identified flies sleeping 2.7h or less as short sleepers and flies sleeping 8h or more as long-sleepers (Figure 1B).

### Disruption of Mitochondrial Complex I causes abnormal sleep

The activity of dopaminergic neurons normally changes across early life, yet the cell-autonomous processes restraining juvenile DA neuron activity are unknown. We reasoned that short-sleep lines recovered from our screen would enrich for LOF conditions causing excess DA neuron activity, including in juvenile flies. Our shortest sleeping hit (2.1 +/− 1.4h of day sleep) was an RNAi line targeting *NDUFAF4*, an assembly factor for Mitochondrial Complex I (MCI) (Figure 1C). Moreover, we uncovered two additional RNAi lines targeting integral MCI subunits (*NDUFB10* and *NDUFS2*) as causing reduced sleep, with 3.9 +/− 1.9h of day sleep for *Th>NDUFB10* IR and 3.6 +/− 1.3h for *Th>NDUFS2* IR flies (Figure 1C). None of these RNAi lines affected waking locomotor activity (Figure 1D). In *Drosophila,* MCI consists of 43 subunits organized into an inner membrane-associated domain and a hydrophilic arm protruding into the matrix(10). The hydrophilic domain initiates electron transport by accepting 2 electrons from reduced nicotinamide adenine dinucleotide (NADH) and shuttling them toward CoQ in the inner mitochondrial membrane. This transfer is coupled to H^+^ translocation against its electrochemical gradient (Figure 1E). NDUFAF4 promotes the assembly of the large holoenzyme complex from smaller subunits(11). NDUFS2 contains an Fe-S cluster that directly participates in the MCI redox cycle and also contributes residues to the CoQ binding site. NDUFB10 encodes an accessory subunit of the membranous arm of MCI whose human ortholog, NDUFB10, is necessary for MCI assembly and function(12, 13). Collectively, the screen uncovered converging lines of evidence that loss of MCI function in DANs causes sleep loss.

### Weak versus strong MCI loss-of-function explains divergent sleep phenotypes

Neurons have tremendous ATP requirements due to the elaboration and maintenance of their neurites, maintenance of their resting membrane potential, and synaptic vesicle cycling. It was therefore surprising to find MCI loss-of-function (LOF) as causing *hyper*arousal, suggesting elevated DAN activity. However, we also recovered an RNAi line targeting the NADH-binding subunit of MCI, NDUFV1, as one of our longest-sleeping flies (8.8 +/− 1.6h of day sleep, Figure 1C), consistent with DAN hypofunction. What accounts for these opposing sleep phenotypes?

First, to validate our screen hits, we repeated the MCI RNAi experiments using additional genetic controls and higher spatial resolution sleep monitoring. We replicated the mature adult day sleep loss seen in *Th>NDUFB10* IR and *Th>NDUFS2* IR flies (Figure 1F–I). Night sleep metrics were unaffected (Supplemental Figure 2A,B). The *Th>NDUFAF4* IR mature adult phenotype was variable but trended toward reduced daytime sleep, with many flies sleeping far less than controls (Supplemental Figure 2D–G). In contrast, the increased daytime quiescence observed in *Th>NDUFV1* IR flies (Figure 1J, K) was significantly correlated with a reduction in waking locomotor activity (Supplemental Figure 2C, Spearman r=−0.45, p=0.03), indicating much of the increase in quiescence can be explained by general hypoactivity. As with the short-sleeping lines, night sleep measures were unaffected in *Th>NDUFV1* IR flies (Supplemental Figure 2B).

We wondered if the short sleeping vs hypoactive *Th>*MCI dopaminergic RNAi phenotypes reflect differential MCI LOF or subunit-specific roles. We expressed UAS-*NDUFV1* IR pan-neuronally (*elav>NDUFV1* IR) and observed a prolonged wandering third instar stage and pupal lethality. By contrast, *elav>NDUFB10* IR, *elav>NDUFS2* IR, and *elav>NDUFAF4* IR flies all survived into adulthood with no visible defects (Table 1). This result suggested either that neurons were differentially sensitive to loss of specific MCI regulators, or the short-sleeping RNAi lines preserved enough MCI activity to sustain flies through pupation. To rule out the former hypothesis, we used pan-neuronal CRISPR to ablate the integral MCI subunits *NDUFB10, NDUFS2*, and *NDUFV1* in neurons, reasoning that the Cas9-induced genetic lesions should cause a stronger LOF than RNAi. Indeed, using a pan-neuronally expressed Cas9 (*elav>Cas9),* we found that somatic neuronal mutagenesis of all three loci was adult lethal: *elav>Cas9; g-NDUFB10* and *elav>Cas9; g-NDUFS2* progeny died as pupae (Table 1). Thus, adult neurons do indeed require *NDUFB10* and *NDUFS2* function to complete pupation, arguing against subunit-specific phenotypes. We thus used quantitative PCR to measure mRNA knock-down efficiency for each RNAi line. In line with previously published findings in muscle using identical RNAi lines(14), we found the short-sleeping MCI RNAi lines depleted their target mRNAs but to a lesser degree than the hypoactive UAS-*NDUFV1* RNAi line (Table 1). These results are consistent with the short-sleeping RNAi lines causing weaker MCI LOF when expressed in neurons.

**Figure 2:**
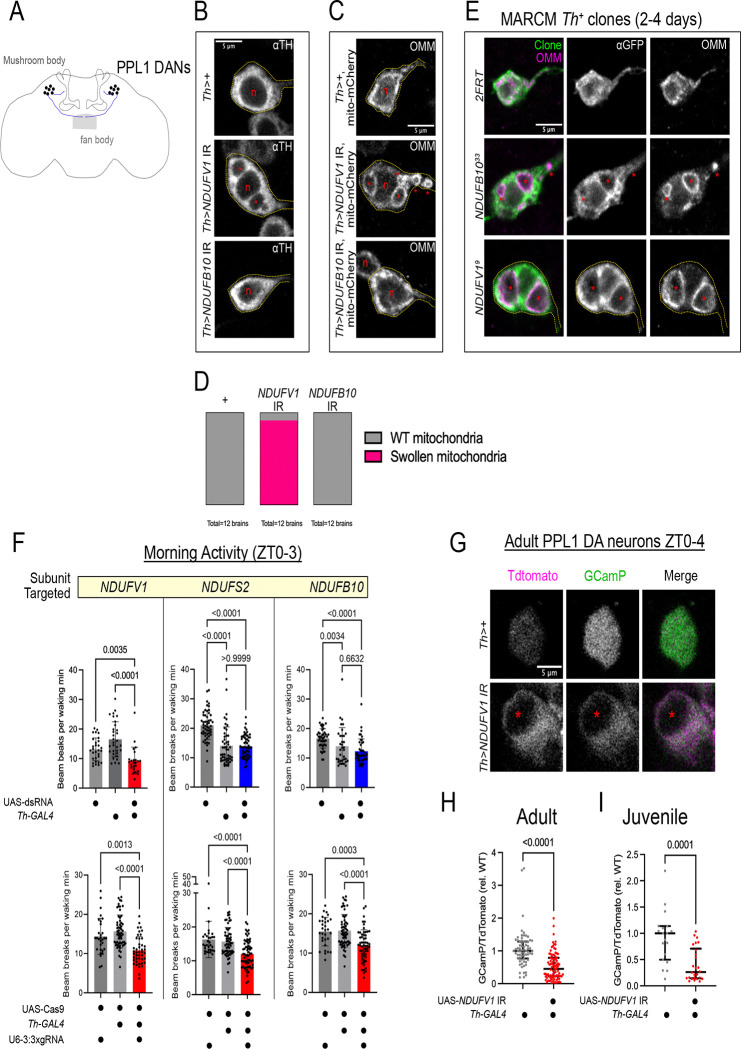
Strong MCI loss-of-function caused dopaminergic hypofunction (A) Paired posterior lateral 1 (PPL1) dopaminergic neurons extend axons to sleep-regulatory regions in the fan body of the central complex and mushroom bodies. (B) Confocal micrographs of the cell bodies of PPL1 DANs labeled by anti-Tyrosine Hydroxylase in control (Th>+), *Th>NDUFV1* IR and *Th>NDUFB10* IR brains. DANs in *Th>NDUFV1* IR brains accumulated cytoplasm-excluding structures (asterisks) around the nucleus (n) not found in control or *Th>NDUFB10* IR brains. (C) PPL1 DANs co-expressing MCI RNAi transgenes and a mito.OMM-mCherry reporter labeling the outer mitochondrial membrane (OMM). In control and *Th>NDUFB10* IR cells, mitochondria in the cell bodies are found in a diffuse reticular network filling the cell body. In *Th>NDUFV1* IR cells, mito.OMM-mCherry labels swollen mitochondria in which the mitochondrial lumens (*) were resolvable. (D) Quantification of the penetrance of swollen mitochondria in PPL1 DANs from control (Th>+), *Th>NDUFV1* IR, and *Th>NDUFB10* IR brains. (E) CD8::GFP-labeled wild-type (*2FRT), NDUFB10*^*33*^*, and NDUFV1*^*9*^ MARCM PPL1 clones expressing mito.OMM-mCherry. MCI null clones for either MCI subunit exhibited swollen mitochondria (asterisks), demonstrating that both NDUFB10 and NDUFV1 subunits are essential for normal dopaminergic mitochondrial morphology. (F) Waking locomotor activity during ZT0–3 for MCI RNAi (top) and MCI somatic CRISPR (bottom) manipulations in DANs. Depressed locomotor activity was observed for only strong MCI loss-of-function manipulations such as *Th>NDUFV1* IR and somatic knock-out of MCI subunits. P values computed using either Kruskal-Wallis tests with Dunn’s multiple-testing comparisons, or Welch ANOVA with Dunnett’s T3 multiple-testing comparisons. (G-I) *ex vivo* imaging of *Th-LexA>TdTomato-2A-GCamP6s; Th-GAL4> NDUFV1* IR adult (H) and juvenile (I) brains. GCamP signal is in green and TdTomato is in magenta. Strong MCI RNAi caused a significant reduction in the GCamP/TdTomato ratio, indicating reduced neuronal activity. Asterisk marks swollen mitochondria. Data from 3 independent experiments N=6 animals per genotype (n~60 cells per genotype in H, n~20 cells per genotype in I). Data are normalized to the median value of the control brains imaged in parallel. P values computed using Mann-Whitney tests.

To address RNAi efficacy differences in DANs more directly, we examined DA neuron morphology in *Th>NDUFB10* IR and *Th>NDUFV1* IR brains, focusing on the PPL1 sub cluster (Figure 2A) given their relevance to sleep/wake behavior (15, 16). Th>*NDUFV1* IR DAN cell bodies contained prominent cytoplasm-excluding structures not found in control or *Th>NDUFB10* IR DAN cell bodies (Figure 2B). These structures corresponded to swollen mitochondria not observed in control or *Th>NDUFB10* IR DANs (Figure 2C,D), indicating that mitochondrial function is more severely compromised in DANs of hypoactive *Th>NDUFV1* IR flies compared with short-sleeping *Th>NDUFB10* IR flies. Our data suggest modest MCI disruption results in reduced sleep, while general hypoactivity emerges as a consequence of severe mitochondrial dysfunction.

### *NDUFB10* is required for ongoing MCI activity and dopaminergic mitochondrial function

Mitochondrial complex I is ubiquitously expressed and supports the ATP demands of all cells in *Drosophila,* complicating functional analysis specifically in adult neurons beyond transgenic RNA interference. To clarify the function of NDUFB10 in dopaminergic mitochondrial function, we used our transgenic guide RNA flies to generate *NDUFB10* and *NDUFV1* germline mutant alleles on an FRT-bearing chromosome (Supplemental Figure 3A, B), allowing for the analysis of genetic mosaics (see Materials and Methods). After recovering non-complementing alleles, we leveraged the essential role for oxidative phosphorylation in normal eye development to prioritize alleles for characterization(17, 18). In somatic eye clones generated using the *EGUF/Hid* approach(19), *NDUFB10*^*33*^ caused small, glassy eyes (Supplemental Figure 3C) similar to clones homozygous for a *NDUFV1* null allele (*NDUFV1*^*9*^) (Supplemental Figure 3C). The *NDUFV1*^*9*^ allele is larval lethal and results from a premature stop codon after G66, truncating the open reading frame upstream of the NADH-binding domain and Fe-S clusters that mediate the catalytic activity of NDUFV1 (Supplemental Figure 3A). Since NDUFV1 initiates electron transfer at MCI from NADH, *NDUFV1*^*9*^ is likely to abolish most MCI activity. *NDUFB10*^*33*^ is also larval-lethal and carries a 2-nucleotide deletion leading to a missense polypeptide N-terminal to a conserved disulfide bridge and lacking the endogenous stop codon (Supplemental Figure 3B, E). Mutations abolishing this disulfide bridge in humans block NDUFB10/NDUFB10 protein import into mitochondria and cause severe neonatal MCI deficiency (20).

In addition, we serendipitously recovered a temperature sensitive allele, named *NDUFB10* ^*ts*^. This allele is an in-frame deletion of 4 amino acids (Y93-P96) in the linker region between the two disulfide-bonded alpha helices (Supplemental Figure 3B,E). *NDUFB10* ^*ts*^ *e*ye clones appeared phenotypically normal at 25°C but were severely disrupted at 29°C (Fig 2 Supplement 1C). Moreover, *NDUFB10* ^*ts*^ failed to complement *NDUFB10*^*33*^ larval lethality at 25°C or higher but could fully rescue *NDUFB10*^*33*^ lethality at 22°C (Supplemental Figure 3D). Based on a high-confidence AlphaFold structure(21) of NDUFB10, Y93-P96 mediate several critical elements of tertiary structure of the disulfide-bonded alpha helices (α2 and α3): E94 in the α2-α3 linker is hydrogen-bonded to K101 in α3; the P96 side chain generates the bend of the linker region and the carbonyl group of its backbone is H-bonded with the conserved H98 side chain in α3 (Supplemental Figure 3E). Lower rearing temperatures could allow the stabilization of the α2 and α3 helices even when these stabilizing elements are lost.

With these genetic tools, we were able to bypass early requirements for essential MCI subunits and directly test functional requirements in adults. First, we measured MCI activity in wild-type and *NDUFB10*^*ts*^ flies reared at 22C during development and shifted to 29C for ~ 1 week. We observed a reduction in MCI activity in the mutant, confirming these mutant alleles reduce enzymatic function (Supplemental Figure 3F). We next generated GFP-labeled *NDUFB10*^*33*^ MARCM clones of PPL1 DA neurons to examine the effect of LOF on DANs specifically (22). To examine adult phenotypes, clones were induced in second instar larvae (48–66h after egg-laying), as this was the latest period we were able to recover *Th+* clones in adult central brains with appreciable frequency. We were able to recover mutant *Th+* clones in 2–4 day old adults with no gross branching defects (Supplemental Figure 3G–I). However, in contrast to wild-type MARCM clones or *Th>NDUFB10* IR cells, *NDUFB10*^*33*^ DANs exhibited swollen mitochondria closely resembling those seen in *NDUFV1*^*9*^ null clones and *Th>NDUFV1* IR cells (Figure 2E). Thus, while *NDUFB10* is essential for mitochondrial function in DANs, Complex I in *Th>NDUFB10* IR flies must retain enough activity to preserve mitochondrial morphology, thereby uncovering an unexpected sleep loss phenotype.

### Genetic dissociation of locomotor and sleep phenotypes caused by MCI-deficiency in DA neurons

In flies, locomotor activity peaks at dawn and dusk (Supplemental Figure 4A–B, grey bars). The peak in morning activity depends on increased post-synaptic DA signal receptivity the morning (23). When a TdTomato-2A-GCamP6s reporter was expressed in wild-type DANs, we observed higher steady-state GCamP/TdTomato ratios in PPL1 DANs during ZT0–3 than ZT4–8 (Supplemental Figure 4C), indicating the peak in morning locomotor activity also coincides with higher intrinsic activity in DANs. *Th>NDUFV1* IR flies exhibited a pronounced depression in the amplitude of morning (Figure 2F) and evening waking activity (Supplemental Figure 4G, H), which we hypothesized is caused by reduced DAN activity. To test this, we expressed the TdTomato-2A-GCamp6s activity reporter under the control of *Th*-LexA along with the UAS-*NDUFV1* IR transgene in DANs. From *ex vivo* preparations of adult brains at ZT0–3, *NDUFV1* knock-down caused a reduction in the steady-state GCamP/TdTomato ratio compared to control cells imaged in parallel (Figure 2G, H). A reduction in cytosolic Ca levels was apparent even in juvenile (1 day old) *Th>NDUFV1* IR; *Th-LexA> TdTomato-2A-GCamP6s* brain preparations (Figure 2I), indicating *NDUFV1* depletion causes DAN hypofunction at or before eclosion. The GCamP cellular phenotype at ZT0–3 is consistent with the behavioral phenotype of reduced morning locomotor activity, suggesting that reduced neuronal activity in *NDUFV1*-depleted DANs is causative.

Importantly, morning and evening activity peaks were unaffected in short-sleeping *Th>NDUFB10* IR and *Th>NDUFS2* IR flies (Figure 2F and Supplemental Figure 4B, G), except for a reduction in locomotor activity after lights-off observed in all MCI perturbations (Supplemental Figure 4H). This suggests that partial MCI LOF does not impair DA release. On the other hand, with *Th*-specific Cas9 (Supplemental Figure 4D–F) to genetically ablate MCI subunits, we observed morning and evening hypoactivity regardless of MCI subunit targeted (Figure 2F and Supplemental Figure 4G, H). Thus, circadian locomotor defects primarily arise when MCI function is severely perturbed.

### MCI inhibition in *Th+* cells causes lifespan defects and neurodegeneration

Because severe MCI LOF causes early toxicity in DANs (Figure 2), we asked whether *Th>NDUFV1* IR flies exhibited lifespan defects. The median survival time at 29C of *Th>NDUFV1* IR flies was 15 +/− 1 days (Supplemental Figure 5A, top). Flies with partial MCI inhibition in DANs (*Th>NDUFB10* IR) also died prematurely but with a median survival time of 20 +/− 2 days (Supplemental Figure 5A, bottom). When PPL1 DANs were counted from aged *Th>NDUFB10* IR flies, we observed a significant reduction compared to genetic controls treated in parallel (Supplemental Figure 5B). Thus, partial MCI LOF leads to an age-dependent loss of DANs, despite the absence of early signs of hypofunction.

### Partial MCI inhibition augments wake-promoting DA signaling primarily during the day

**Figure 3:**
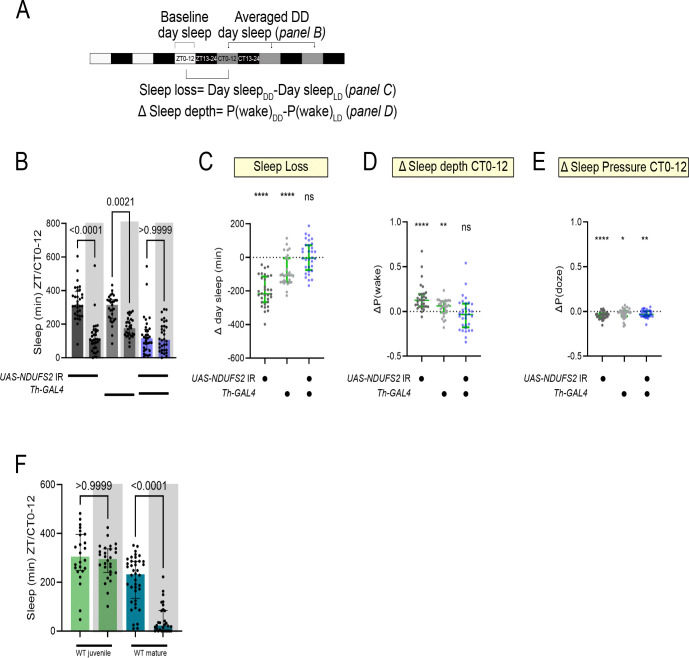
Disinhibition of DA signaling by withdrawal of the light cue modifies wild-type sleep, but not MCI-deficient, flies. (A) Light schedule for assaying sleep phenotypes in constant darkness. (B) Comparison of baseline day sleep with the light cue to subjective day sleep without the lights on (shaded, averaged across 3 successive days). *Th>NDUFS2* IR flies still exhibited short sleep under constant darkness. Control flies exhibited significant sleep loss after withdrawal of the light cue, which was not observed in *Th>NDUFS2* IR flies. (C) Change in day sleep [Day sleep(DD)-Day sleep (LD)] plotted for each fly in the experiment in B. Values < 0 (dotted line) indicate loss of sleep. *NDUFS2* knock-down abrogated sleep loss due to withdrawal of the light cue. (D) Change in P(wake) [P(wake)(DD)-P(wake)(LD)] plotted for each fly in panel B. Sleep loss upon withdrawal of the light cue was driven by an increase in P(wake)--an effect attenuated *NDUFS2* knock-down. (E) Change in P(doze) [P(doze)(DD)-P(doze)(LD)] plotted for each fly in panel B. Withdrawal of the light cue caused a slight but significant decrease in P(doze) that was not affected by *NDUFS2* knock-down. P values calculated by Wilcoxon signed rank tests (* P<0.05, ** P<0.01, **** p<0.0001 compared to Δ=0). (F) Sleep during ZT0–12 or CT0–12 (shaded) for wild-type juvenile and mature flies. While mature flies showed a strong sleep suppression without the morning light cue, juvenile flies were more refractory. All graphs show mean with standard deviation.

The day-specific sleep loss seen in mature *Th>NDUFB10* IR and *Th>NDUFS2* IR flies suggests the sleep-promoting effect of MCI is either light-dependent or clock-dependent. When *Th>NDUFB10* IR and *Th>NDUFS2 IR* flies were shifted to constant darkness, daytime sleep loss persisted (Figure 3A and B, Supplemental Figure 6A, blue bars), indicating the phenotype is not light-dependent.

Light dampens the wake-promoting effect of DA(24). When the morning light cue was removed, control flies showed a marked reduction in subjective daytime sleep (CT0–12) relative to the previous day with the light cue (ZT0–12) (Figure 3A–C and Supplemental Figure 6A,B). Sleep loss during CT0–12 was driven principally by an increased probability of flies transitioning out of sleep [measured by P(wake)(25)], indicative of shallower sleep. On the other hand, the probability of transitioning from wake to sleep [P(doze)] was only negligibly reduced or unchanged during CT0–12 (Figure 3D–E and Supplemental Figure 6C,D). Sleep loss during CT12–24 was more variable and modest, suggesting the arousal cue normally inhibited by light is strongest during the day (Supplemental Figure 6E,F). Since DA promotes arousal largely by increasing P(wake)(25), an elevated P(wake) upon withdrawal of the light cue is consistent with disinhibition of DA-mediated arousal. Consistent with this interpretation, wild-type juvenile *iso*^*31*^ flies (which have a relatively low DAN activity) were notably refractory to sleep loss compared to mature adults (Figure 3F) in this same light schedule. Thus, we suggest that by removing the light cue, wild-type flies become sensitized to DA-mediated arousal that is strongest during CT/ZT0–12.

Interestingly, in *Th>NDUFS2 IR* and *Th>NDUFB10 IR* flies, sleep duration during ZT0–12 (i.e. lights on) closely resembled sleep in control flies during the same period under constant dark; moreover, knock-down flies failed to exhibit further sleep loss when the lights did not come on (Figure 3B and Supplemental Figure 6A). From these experiments, we suggest that partial MCI inhibition in DANs mimics the effect (i.e. augmented DA signaling) of withdrawing the light cue in wild-type flies. Since the arousal-promoting DA cue is strongest during the day, this is a likely explanation for the stronger daytime phenotypes across our MCI perturbations.

### Partial MCI inhibition reduces juvenile sleep depth

**Figure 4:**
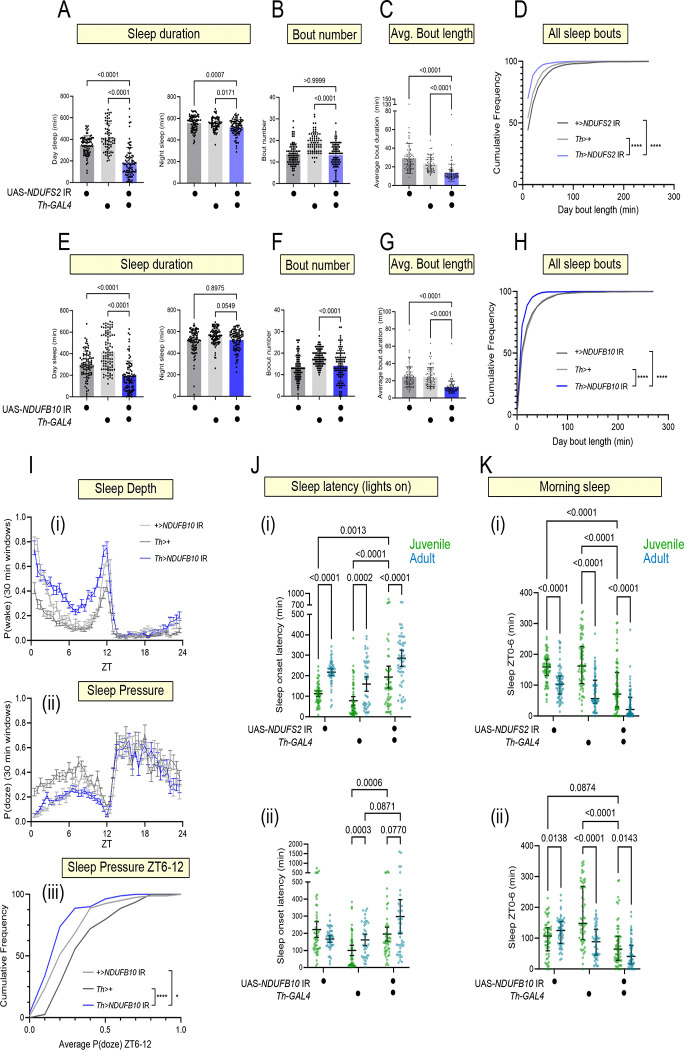
Partial MCI loss-of-function impaired juvenile sleep depth leading to fragmented sleep (A) Day and night sleep duration in juvenile *Th>NDUFS2* IR flies (blue) and controls (grey). (B-D) Day sleep metric in juvenile *Th>NDUFS2* IR flies and controls. Sleep loss during the day was caused by reduced sleep bout duration (C,D, **** P<0.0001). (E) Day and night sleep duration in juvenile *Th>NDUFB10* IR flies (blue) and controls (grey). (F-H) Daytime sleep metric of juvenile *Th>NDUFB10* IR flies and controls. Sleep loss was caused by reduced sleep bout duration (G,H, **** p<0.0001). P values computed using either Kruskal-Wallis tests with Dunn’s multiple-testing comparisons or Welch ANOVA tests with Dunnett’s T3 multiple-testing comparisons. (I) P(wake) (i) and P(doze) (ii) calculated across 30 min intervals (mean +/− SEM) across ZT0–24 in *Th>NDUFB10* IR juvenile flies (blue) and controls (grey). Knock-down flies showed consistently increased in P(wake) during ZT0–12 compared to control flies, indicating impaired sleep depth. P(doze) was only significantly reduced in *Th>NDUFB10* IR juvenile flies during ZT6–12 (iii). P values computed using Kruskal-Wallis tests with Dunn’s multiple-testing comparisons. (J) Time to the first sleep bout after ZT0 (sleep latency) for juvenile (green) and mature (blue) *Th>NDUFS2* IR flies and genetic controls (i) and *Th>NDUFB10* IR flies and genetic controls (ii). Robust MCI activity was required to maintain relatively short sleep latencies observed in juvenile flies. (K) Sleep ontogeny phenotypes in *Th>NDUFS2* IR (i) and *Th>NDUFB10* IR (ii) flies as measured by sleep duration during ZT0–6. Juvenile flies (green dots) across all genotypes exhibited greater sleep during the first part of the day compared to their mature counterparts (blue dots), indicating sleep maturation is not impaired by partial MCI LOF. P values in J and K computed using Two-way ANOVA with mix-effects model and Tukey’s multiple comparisons tests

DANs are normally less active in a juvenile fly(5), facilitating increased sleep during early life. The experiments above suggest partial MCI inhibition in juvenile DANs might increase their activity, in turn interfering with juvenile sleep drive. We began by assessing sleep parameters in 1 day-old *Th>NDUFS2* IR, *Th>NDUFB10* IR, and *Th>NDUFAF4* IR juvenile flies under normal LD conditions. All three knock-down conditions exhibited significant sleep loss during the day (Figure 4A, E and Supplemental Figure 7A). Nighttime sleep duration was either unaffected (*Th>NDUFB10 IR*, *Th>NDUFAF4 IR*) or very mildly affected (*Th>NDUFS2 IR*) (Figure 4A,E and Supplemental Figure 7A). Juvenile flies experience deeper sleep compared to mature adult flies because of reduced sleep-to-wake transitions, resulting in longer sleep bouts in juvenile flies (26). Notably, whereas mature *Th>NDUFS2* IR, *Th>NDUFB10* IR, and *Th>NDUFAF4* IR flies exhibited no consistent change in sleep bout length relative to genetic controls (see Figure 1 and Supplemental Figure 2), juvenile flies exhibited shorter sleep bouts during both ZT0–12 and ZT12–24 across all 3 MCI manipulations (Figure 4C,D, G, H and Supplemental Figure 7C, D, E–G). Thus, although sleep loss is not stage-specific, partial MCI LOF causes juvenile-specific sleep fragmentation.

Sleep fragmentation suggests an increased likelihood of transitioning from sleep to wake. Indeed, knock-down flies for all partial MCI manipulations exhibited elevated P(wake) during ZT0–12 (Figure 4I (i) and Supplemental Figure 7H, I). The effect of partial MCI LOF on P(doze) was difficult to assess because of variability across controls, but we did observe reduced P(doze) largely restricted to ZT6–12 for some RNAi conditions (Figure 4I(ii)–(iii)). From these analyses, we conclude that partial MCI LOF in DANs interferes with juvenile sleep principally by compromising sleep depth.

Juvenile flies are quicker than their adult counterparts to initiate sleep after the peak in morning activity following lights-on (ZT0) and lights off (ZT12)(5) (Figure 4C, D). Sleep latency in the juvenile *Th>NDUFS2* IR and *Th>NDUFB10* IR flies more closely resembled those of mature adult control flies (Figure 4C,D), consistent with a diminution of juvenile sleep drive. Sleep latency after lights off was unaffected by partial MCI inhibition (Supplemental Figure 7J). Morning locomotor activity was unaffected (Supplemental Figure 7K, suggesting these flies did not take longer to fall asleep merely because they were hyperactive. Rather, partial MCI inhibition in DA neurons specifically interferes with daytime sleep-related behaviors needed for high juvenile sleep drive.

The increased sleep fragmentation and sleep latency seen upon partial MCI inhibition collectively indicate a disruption of normal juvenile sleep drive. Do changes in dopaminergic MCI activity in DANs explain why juvenile flies sleep more than adults? During sleep maturation from juvenile to mature adulthood, the distribution of sleep throughout the light period changes, including more time spent asleep in the morning(5). To determine whether sleep maturation was perturbed by partial MCI inhibition in DANs, we compared sleep latency and sleep duration during ZT0–6 in 1 day old vs 6 day old flies. Across the first week of adult life, control flies showed a progression from low to high sleep latency (Figure 4C,D), as well as a progression from higher to lower morning sleep duration (Figure 4E,F). These trends were preserved upon partial MCI inhibition in in DANs (Figure 4C–F). Therefore, while partial MCI inhibition does impair juvenile sleep drive, it does not prevent the normal ontogenetic sleep change across the first week of adult life.

### Depletion of reduced CoQ in DANs causes sleep loss

**Fig 5:**
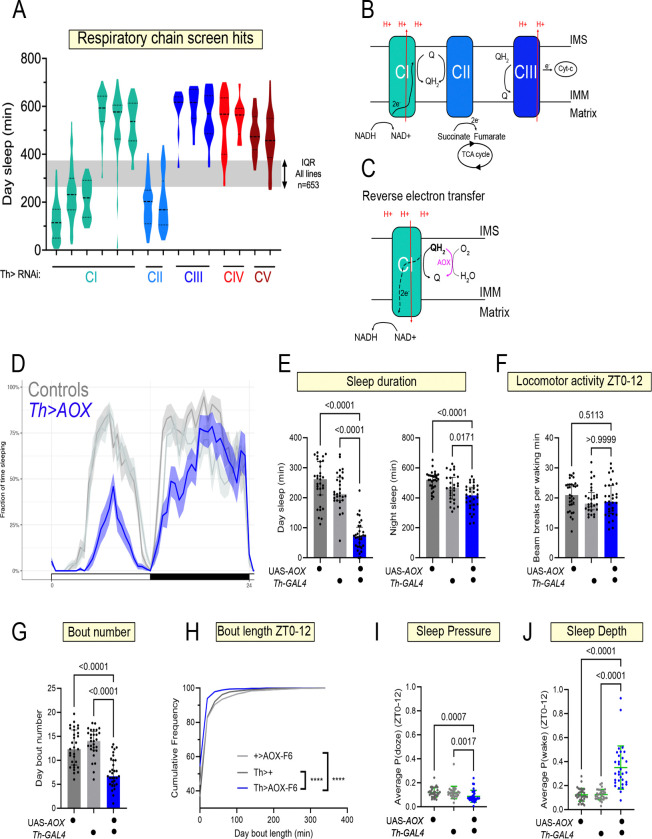
The CoQ redox state of dopaminergic neurons influences sleep (A) Summary of daytime sleep phenotypes for all OXPHOS pathway screen hits. Grey shading reflects the interquartile range (IQR, the middle 50% of phenotypes) for all RNAi lines tested during the screen. While targeting any part of the respiratory chain can cause increased quiescence (due to reduced locomotor defects), only manipulations upstream of the CoQ pool (MCI and MCII) caused short sleep. (B) Schematic of respiratory complexes affecting the CoQ redox state. Reduced MCI and MCII activity both cause depletion of CoQH_**2**_. (C) Electron transfer at MCI can run in reverse, using electrons from CoQH_**2**_ to regenerate NADH at the expense of the PMV. The Alternative Oxidase (AOX) can directly deplete CoQH_**2**_ by transferring its reducing equivalents to oxygen. (D) Sleep per 30 min windows (average with 95% confidence interval shaded) of *Th>AOX* mature adult flies (blue) and genetic controls (grey). Depletion of CoQH2 was strongly wake-promoting, particularly during the ZT0–12. (E) Sleep duration during day (ZT0–12) and night (ZT12–24) for *Th>AOX* flies and genetic controls. Sleep loss was most pronounced during ZT0–12. (F) Waking locomotor activity during ZT0–12 for *Th>AOX* flies and genetic controls. Sleep loss was not associated with hyperactivity. (G) Number of sleep bouts for *Th>AOX* flies and genetic controls. AOX-overexpressing flies showed reduced sleep initiation. (H) Cumulative frequency plot of the durations of daytime sleep bouts for *Th>AOX* flies and genetic controls. AOX-overexpressing flies showed significantly reduced sleep bout duration. (**** p<0.0001. Kruskal-Wallis test with Dunn’s multiple testing comparisons) (I) P(doze) and (J) P(wake) averaged across ZT0–12 for *Th>AOX* flies and genetic controls. AOX overexpression affected both P(doze) and P(wake), but the effect on P(wake) was largest, indicating sleep loss in *Th>AOX* flies was driven largely by reduced sleep depth. P values computed using Kruskal-Wallis tests with Dunn’s multiple-testing comparisons.

Our behavioral screen identified numerous hits in oxidative phosphorylation (OXPHOS) pathway components spanning MCI to MCV. Most of these hits exhibited increased quiescence (Figure 5A), reflecting general hypoactivity due to reduced ATP synthesis (Supplemental Figure 8A,B). However, only manipulations upstream of the reduced CoQ (CoQH_2_) pool caused sleep loss. In addition to the aforementioned MCI manipulations, we identified *Th>sdhAF4* IR flies as short sleepers (Figure 5A). SDHAF4 is an assembly factor for Succinate Dehydrogenase/Mitochondrial Complex II, which oxidizes the TCA cycle intermediate succinate and passes those electrons to CoQ. SDHAF4 is essential for MCII holoenzyme function(27) and both RNAi lines comparably depleted *sdhAF4* mRNA (Supplemental Figure 8C), strongly indicating the specificity of the phenotype. In juvenile flies, *sdhAF4* knock-down in DANs caused sleep loss during ZT0–12 (Supplemental Figure 8D) through an increased likelihood of transitioning from sleep to wake (Supplemental Figure 8E)--a phenotype identical to partial MCI inhibition. These results strongly suggest that, in the context of disruptions to the OXPHOS pathway, depletion of CoQH_2_ is necessary to cause sleep loss.

To deplete CoQH_2_ levels without interfering with the composition of the transport chain, we expressed the Alternative Oxidase from *Ciona intestinalis* which depletes CoQH_2_ by transferring its excess electrons directly to oxygen to generate water(28). Adult *Th>AOX* flies showed a pronounced sleep loss during the day and early part of the night, driven by both reduced bout number and bout length (Figure 5D, E, G, H). Locomotor activity was not affected (Figure 5F), indicating sleep loss is not caused by hyperactivity. Though P(doze) (Figure 5I) and P(wake) (Figure 5J) were both affected by AOX overexpression, the most pronounced effect was an elevated P(wake), indicating that *Th>*AOX flies lose sleep principally by a reduction in sleep depth, consistent with gain of DAN function. Importantly, sleep loss was reproduced using two independent UAS-AOX insertions (Supplemental Figure 8F–J), ruling out insertion-site-specific position effects. These results indicate that maintenance of high levels of CoQH_2_ in the dopaminergic respiratory chain promotes sleep.

### The redox state of Coenzyme Q regulates juvenile DA neuron activity

**Fig 6:**
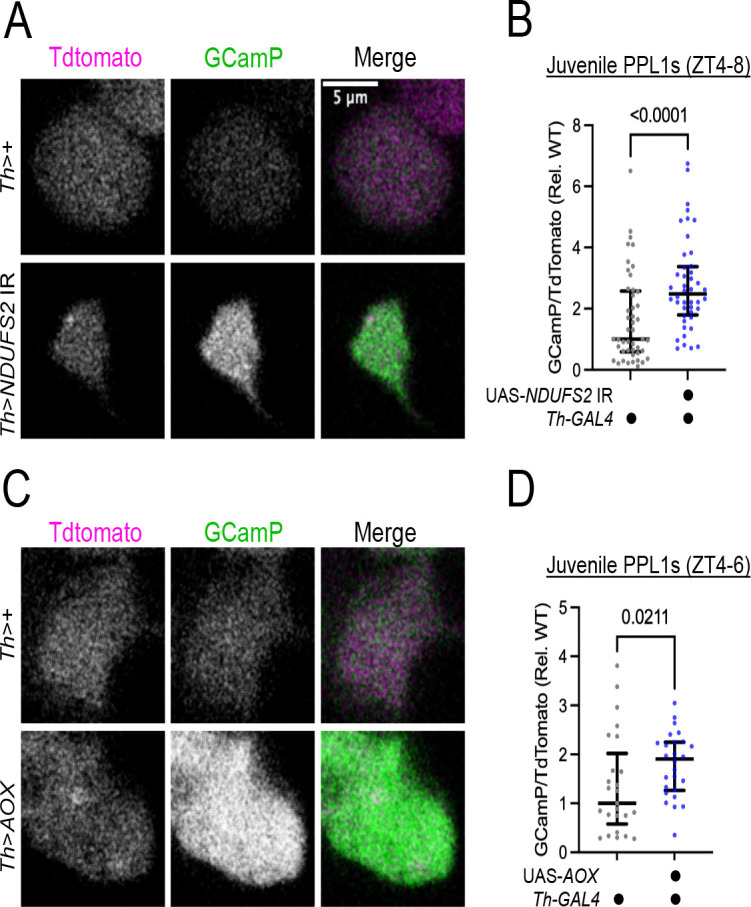
Depletion of UQH2 increases dopaminergic neuron activity A) *Ex vivo* imaging of PPL1 DANs from *Th-LexA>TdTomato-2A-GCamP6s; Th-GAL4>+ attP2* (top) and *Th-LexA>TdTomato-2A-GCamP6s; Th-GAL4>NDUFS2* IR (bottom) juvenile brains. TdTomato is shown in magenta and GCamP in green. (B) Quantification of the GCamP/TdTomato ratios (median and interquartile range) in the juvenile PPL1 DAN cell bodies during ZT4–8. Ratios are normalized to the median of the control brains imaged in parallel. Partial MCI inhibition caused an increase in cytosolic Ca^**++**^ levels, in contrast to the reduction seen with severe MCI RNAi. P value computed by a Mann-Whitney t-test. (C) *Ex vivo* imaging of PPL1 DANs from *Th-LexA>TdTomato-2A-GCamP6s; Th-GAL4>+* (top) and *Th-LexA>TdTomato-2A-GCamP6s; Th-GAL4>AOX-F6* (bottom) juvenile brains. (D) Quantification of GCamP/TdTomato ratios (median and interquartile range) in juvenile PPL1 DAN cell bodies during ZT4–6. AOX overexpression elevated cytosolic Ca^**++**^ levels in DANs. P value computed by a Mann-Whitney t-test.

Because multiple, independent genetic perturbations depleting CoQH2 in DANs caused sleep loss, and since excessive DA release also cause sleep loss(29), we hypothesized that the CoQ/CoQH2 redox state influences DA neuron activity. To this end, we measured cytosolic Ca levels normalized to an activity-independent TdTomato reporter in juvenile DANs with or without expression of UAS-*NDUFS2* IR. During the afternoon (ZT4–8), we observed an increase in steady-state Ca++ levels in *NDUFS2*-deficient DANs (Figure 6A, B), coinciding with the period of increased P(wake) (Supplemental Figure 7H). This is in stark contrast to strong MCI inhibition using *Th>NDUFV1* IR, in which we observed a reduction in cytosolic Ca++ levels (Figure 2I). A similar increase in cytosolic Ca++ levels was observed for *AOX*-expressing PPL1 DANs from juvenile flies (Figure 6C, D). Thus, depletion of COQH2, as opposed to MCI perturbation per se, accounts for the increased cytosolic Ca++ seen in *Th>NDUFS2* IR DANs. From these experiments, we conclude that the redox state of Coenzyme Q regulates juvenile DA neuron activity.

## Discussion

Sleep undergoes a coordinated maturation across early life, yet the cellular mechanisms that shape this transition remain unclear. Here, we identify mitochondrial Complex I and coenzyme Q redox state in dopaminergic neurons as key regulators of sleep drive. Transcriptomic profiling revealed elevated expression of metabolic and oxidative phosphorylation genes in juvenile dopaminergic neurons, suggesting an elevated demand for metabolic gene expression in juvenile flies. While we corroborated the known requirement for MCI in the general maintenance of dopaminergic neuron function, our work revealed a surprising role for the CoQ redox state in promoting sleep by regulating DAN activity. High juvenile sleep drive is perturbed when CoQH_2_ levels are depleted, which results in elevated DA neuronal activity. Since CoQH_2_ is the source of reducing equivalents for reverse electron transfer at MCI, our data are consistent with a model in which RET at MCI functions physiologically to limit DA neuron activity. While not restricted to juvenile DANs, this mechanism is nevertheless essential at this stage to promote high sleep drive.

Disturbed mitochondrial energetics is a primary cause of dopaminergic degeneration leading to Parkinsonism. In particular, both environmental and genetic drivers of the motor symptoms of Parkinsonism converge on reduced MCI activity(30). There is mounting evidence that sleep disturbances may function as early harbingers, and even drivers, of later neurodegeneration(31). We identified a surprising sleep phenotype with partial disruption of MCI activity in DANs. Rather than causing an intermediate locomotor phenotype between wild-type and severe MCI LOF, we observed sleep loss. While sleep loss was not age-specific, we did observe a juvenile-specific sleep fragmentation phenotype caused by increased transitions from sleep to wake. This phenotype is consistent with the known effect of augmented DA signaling, and calcium imaging results are consistent with partial MCI LOF causing elevated DAN activity. Interestingly, dopaminergic-specific ablation of MCI activity in mice causes sleep fragmentation before the onset of motor symptoms and neuron loss(32). Thus, hyperactivity of DANs in early life, rather than hypofunction, might represent an early prodromal symptom of DAN degeneration. Importantly, augmented DAN activity cannot be explained merely by a collapse of mitochondrial function and resultant ATP depletion, since (i) mitochondrial morphology was intact in DANs with partial MCI LOF and (ii) ATP depletion by knock-down of ATPase subunits caused severe locomotor deficits identical to strong MCI LOF. Rather, our work suggests that subtle alterations to the CoQ/CoQH2 redox state in early life influence sleep before neuron loss. Specifically, we find through both MCI-dependent and MCI-independent genetic manipulations that a high CoQ/CoQH2 ratio in DANs is wake-promoting. Since CoQH2 is the source of electrons for reverse electron transfer (RET) at MCI, our results suggest that dopaminergic MCI maintains a basal level of RET to *limit* neuronal activity. Inhibition of RET results in hyperactive DANs, leading to hyperarousal and sleep fragmentation. Thus, early-onset defects in RET and the related sleep disturbances could presage later, more severe deficits in MCI dysfunction accompanying neurodegeneration.

MCI activity is essential for the maintenance of DAN function(33). Our experiments involving strong MCI LOF corroborate the importance of MCI for normal dopaminergic mitochondrial function but indicate an indispensable role during DAN development. With RNAi manipulations, we observed DAN hypoactivity as early as 1 day old juvenile flies. When *NDUFB10* and *NDUFV1* null clones were induced late in *Drosophila* dopaminergic neurogenesis (second instar larvae), mitochondrial dysfunction (as read out by mitochondrial swelling) was apparent as early as ~2 days after eclosion. Thus, *Drosophila* DANs absolutely require MCI activity during metamorphosis or in juvenile adults. Unlike some neuronal sub-types in *Drosophila (34, 35)*, DANs therefore lack compensatory mechanisms to circumvent mitochondrial dysfunction in early life. Interestingly, the neuronal projections of MCI null clones were indistinguishable in early adult flies. Therefore, normal neuronal morphology appears to be established even in DANs carrying null mutations in essential MCI subunits. Future work will address the proximal developmental causes leading to early DA hypoactivity with strong MCI loss of function, perhaps defining subtle pathophysiological mechanisms that arise before frank neurodegeneration in genetic Parkinsonism.

How might alterations in CoQ/CoQH_2_ redox state increase dopaminergic neuron activity? One possibility is that partial inhibition of MCI and RET produces modest changes in redox signaling that enhance neuronal excitability. In neurons, low-level increases in reactive oxygen species (ROS) can potentiate activity by modulating voltage-gated ion channels, intracellular Ca^2+^ handling, or neurotransmitter release(36–38), whereas more severe or sustained redox stress leads to mitochondrial damage and neuronal hypoactivity(38). Alternatively, changes in CoQ redox balance may influence dopaminergic output through metabolic signaling independent of oxidative damage. Subtle shifts in mitochondrial membrane potential, NAD^+^/NADH ratios, or ATP availability at presynaptic sites could increase release probability or excitability(39, 40). Such mechanisms may be particularly relevant in juvenile dopaminergic neurons, which exhibit elevated metabolic gene expression and may rely on tightly regulated redox states to restrain wake-promoting output.

These findings add to a growing body of work implicating mitochondria as key regulators of sleep need. Previous studies have linked broad mitochondrial dysfunction and mitochondrial dynamics to changes in sleep duration and architecture, including within defined sleep-promoting neuronal populations(41, 42). Our work highlights a mechanism in which subtle perturbations of MCI and the redox state of CoQ selectively modulate dopaminergic output to regulate sleep drive. By dissociating redox-dependent signaling from ATP depletion or mitochondrial collapse, our results support a model in which mitochondrial redox processes function as signaling nodes within sleep–wake circuits. This regulation is most apparent during juvenile periods, suggesting that mitochondrial function contributes to the developmental calibration of arousal systems. Thus, the same mitochondrial processes involved in late-stage dopaminergic resilience might also shape the maturation of sleep, linking sleep early in life to how these neurons age. More broadly, these findings are consistent with an emerging idea that sleep phenotypes may serve as sensitive indicators of early mitochondrial dysfunction, revealing circuit-specific vulnerabilities that precede overt neurodegeneration.

## Materials and Methods

### Cell sorting and RNAseq

Juvenile (0–1 day old) and mature (6–9 day old) *Th*>TdTomato brains were dissected in ice-cold Schneider’s medium. Brains were first enzymatically dissociated in Papain (100U/ml) suspended in 1X PBS and supplemented with 0.18U/ml Liberase^™^ cocktail (Roche). Dissociation was carried out for 50 min at room temperature with nutating. Dissociated brains were rinsed three times in cold Schneider’s to stop dissociation. Schneider’s medium was replaced by 2% Bovine Serum Albumin in 1XPBS. Mechanical trituration was performed by first pipetting the brains up and down 100% in P200 pipette tips pre-coated with fetal bovine serum; then, dissociated tissue was passed 20 times through a 3cc gauge needle pre-coated with fetal bovine serum. The suspension was then transferred to FACS tubes kept on ice. DAPI was added to label dead cells. In parallel, +>TdTomato brains were treated in parallel to gate TdTomato signal during FACS sorting. Sorting was performed at the Penn Cytomics core using an Aria cytometer equipped with a 450 nm UV and 582 nm lasers and a 100 um nozzle. FACSDiva (v8.0.1) was used to analyze data. Dopaminergic cells were gated by low DAPI and high TdTomato signal. Cells were sorted directly into a lysis buffer for the SMART-seq HT kit (Takara Biosciences) and flash frozen on dry ice. Library preparation and RNA sequencing was performed using the Illumina NGS platform by Admera Health (South Plainfield NJ, USA) to obtain paired-end 150 bp reads. Trim Galore (v0.4.4_dev) and Cutadapt (v2.4) were used to remove Adapter Sequences from the reads. Salmon (v1.10.1) was used to quantify transcript read counts against transcripts from v110 of the Ensembl Drosophila Melanogaster annotation. Using R (v4.3.1), the Salmon output was summarized to gene-level quantifications using the tximeta package (v1.18.3). DESeq2 (v1.40.2) was used to perform differential expression analysis between the juvenile and mature samples on the unnormalized read counts.

### RNAi screen

Virgin *Th-GAL4* females were matted to males from the Transgenic RNAi Project lines carrying UAS-dsRNAs or UAS-shRNAs carried in VALIUM10, VALIUM20, or VALIUM22 vectors(43, 44). 16 mated UAS-dsRNA/+; *Th-GAL4/+* females were transferred into glass tubes containing 5% sucrose in 2% agar as a food source and loaded into DAM2 activity monitors (Trikinetics, Princeton, MA, USA). Sleep was assayed under 12h:12h light:dark cycles in 6–9 day old flies. Each RNAi line was tested in 2 independent biological runs. Average sleep duration for ZT0–12 was used to identify short- and long-sleepers.

### Sleep assays

Flies were maintained in a 12h:12h light:dark cycle throughout their lifecycle. Virgin females were used for juvenile (1 day post eclosion) and mature adult (5–7 days post eclosion) sleep assays. Flies were anesthetized using CO2 and transferred into glass tubes containing 5% sucrose in 2% agar as a food source. DAM5H activity monitors (Trikinetics, Princeton, MA, USA) were used to track locomotor activity in 12h:12h light:dark cycles at 25C and ~60% relative humidity using Percival incubators. All sleep assays were performed in the same incubator throughout. Activity count data were analyzed using the Rhethomics pipeline(45) in R for most sleep metrics (duration, bout number, bout length, activity index). Pwake and Pdoze were calculated using the Sleep and Circadian Matlab Analysis Program (SCAMP)(46). In all analyses, bouts of inactivity >/= 5 min were used as the standard definition of sleep. Sleep latency times were calculated by manually scoring the time to the first sleep bout (inactivity for >/= 5 min) directly from raw activity counts.

### Fly husbandry

Fly crosses were reared on standard laboratory media (LabExpress) at 25C on a 12h:12h light:dark cycle under ~60% relative humidity. Below are listed lines specifically used in the figures.

**Table:** 

Fly Strain Used	Purpose	Source
*w^1118^, iso^31^*	Genetic background for GAL4 drivers	Kayser et al (2014) (?)
*w^1118^, iso^31^;; P{Th-GAL4}*	Directed expression in dopaminergic neurons	Kayser et al (2014)
*y^1^, w^1^;; P{Th-GAL4}/TM6B*	Directed expression in dopaminergic neurons; used to validate long-sleeping RNAi lines	This work
*w^1118^, elav^c155^-GAL4*	*elav-GAL4* pan-neuronal GAL4 driver	Chakravarti-Dilley et al (2018)
w*;; P{UAS-TdTomato}	FACS sorting of Dopaminergic neurons	Bloomington Stock Center #36328
y^1^v^1^;; P{TRIP.HM05053}attP2	*NDUFAF4* dsRNA expression	Bloomington Stock Center (#28567)
*y^1^v^1^;; P{TRIP.HM05059}attP2*	*NDUFS2* dsRNA expression	Bloomington Stock Center (#28573)
y^1^v^1^;; P{TRIP.JF03271}attP2	*NDUFB10* dsRNA expression	Bloomington Stock Center (#29592)
y^1^ sc* v^1^ sev^21^;; P{TRIP.HMS01590}attP2	*NDUFV1* dsRNA expression	Bloomington Stock Center (#36701)
y^1^, sc* v^1^, sev^21^; P{TRiP.GL01558}attP40	*sdhAF4* dsRNA expression	Bloomington Stock Center (#43213)
*y^1^v^1^;; P{TRIP.HMS02061}attP2*	*sdhAF4* dsRNA expression	Bloomington Stock Center (#80441)
y^1^v^1^;; P{CaryP}attP2	Negative control for RNAi experiments	Bloomington Stock Center (#36303)
y^1^v^1^; P{CaryP}attP40	Negative control for RNAi experiments	Bloomington stock center (#36304)
*w^1118^, elav-GAL4^c155^;; P{UAS-Cas9.P}attP2*	Pan-neuronal Cas9 expression	Cas9 source from BDSC#54592
P{nos-PhiC31-NLS} y^1^ sc* v^1^ sev^21^; P{CaryP}attP2	Site-specific recombination of pCFD5-multiplex gRNA constucts	Bloomington Stock Center (#25710)
y^1^sc* v^1^ sev^21^;; Dr,e /TM3 Sb	Balancing of multiplex gRNA transgenes	Bloomington Stock center (#32261)
y^1^sc* v^1^ sev^21^;; U6-3: P{3Xg-NDUFV1} attP2	Somatic mutagenesis of *NDUFV1*	This work
y^1^sc* v^1^ sev^21^;; U6-3: P{3Xg-NDUFB10} attP2	Somatic mutagenesis of *NDUFB10*	This work
y^1^sc* v^1^ sev^21^;; U6-3: P{3Xg-NDUFS2} attP2	Somatic mutagenesis of *NDUFS2*	This work
*y^1^, M{Act5C-Cas9.PRFP-}ZH-2A, w^1118^, DNAlig4^169^*	Confirmation of gRNA efficacy	Bloomington stock center (#58492)
*y^1^w^1^; UAS-GFP.S65T; gRNA-GFP{pACMG(2.1)2}[D42-2A.VK27]/TM2, P{w-,GMR-Hid}*	Cas9 tester strain for *elav>Cas9* and *Th>Cas9*	Source of EGFP BDSC #1521;gRNA-GFP a gift from Chun Han (Cornell)(47)
*w^1118^, iso^31^; P{UAS-Cas9.P2}attP40/CyO*	Cas9 source back-crossed 8 generations onto *iso31* background	Source of Cas9 BDSC #67079
y^1^, w*;;P{Th-GAL4}, P{UAS-mito.OMM-mCherry}/TM6B	Mitochondrial morphology in DANs	Source mitochondrial reporter BDSC#66533
y^1^, M{nos-Cas9.P}ZH-2A, w*; FRT40A{neoR}, FRT42B{w+}	Starter stock for germline mutagenesis	Source of FRT chromosome BDSC #8217; source of Cas9 BDSC #54591
*w^1118^; Df(2L)Exel8017/CyO*	Deficiency uncovering *NDUFV1;* used in allele screen	BDSC (stock obsoleted)
*w^1118^; Df(2L)ED4559/SM6a, Cy*	Deficiency uncovering *NDUFB10;* used in allele screen	Bloomington Stock center #24121
*w^1118^; NDUFV1^9^ FRT40A/CyO*	Null allele for *NDUFV1;* back-crossed onto *iso31* background for >5 generations	This work
*w^1118^; NDUFB10^33^ FRT40A/CyO*	Null allele for *NDUFB10;* back-crossed onto *iso31* background for >5 generations	This work
*w^1118^; NDUFB10^ts^FRT40A/CyO*	conditional allele for *NDUFB10;* back-crossed onto *iso31* background for >5 generations	This work
*w^1118^; 2FRT*	Parental 2FRT chromosome back-crossed onto *iso31* for >5 generations	This work
*w^1118^; sna^Sco^/Cyo, P{wee-P.ph0}Bacc*	Source of green balancer for genomic PCR analysis of mutants	Mixed source
y^1^, w*; P{GMR-hid}G1 FRT40A, l(2)CL-L^1^/CyO; P{GAL4-ey.H}SS5, P{UAS-FLP.D}JD2	EGUF/Hid mitotic clones	Bloomington Stock Center #76594
P{hsp-FLP}12 y^1^, w*, P{UAS-mCD8:GFP}; P{tubP:GAL80} FRT40A{neoR}/CyO	MARCM stock for 2L clones	Sources: BDSC #28832, #5192
P{hsp-FLP}12 y^1^, w*,; 2FRT/CyO; P{Th-GAL4}, P{UAS-mito.OMM-mCherry}/TM6B	MARCM-ready stock for wild-type clones	This work
P{hsp-FLP}12, y^1^, w*; NDUFV1^9^ FRT40A/CyO; P{Th-GAL4}, P{UAS-mito.OMM-mCherry/TM6B	MARCM-ready stock for *NDUFV1^9^* allele	This work
P{hsp-FLP}12, y^1^, w*; NDUFB10^33^ FRT40A/CyO; P{Th-GAL4}, P{UAS-mito.OMM-mCherry}/TM6B	MARCM-ready stock for *NDUFB10^33^*	This work
y^1^, w*, P{nos-PhiC31-nls}; P{y^+^, CaryP}attP40	Injection background for chromosome 2 GCamP reporter	Rainbow transgenics (Camarillo, CA, USA)
y^1^ w* P{nos-phiC31-NLS};; PBac{y[+]-attP-9A}VK00027	Injection background for chromosome 3 GCamP reporter	Bloomington stock center #35569
*w^1118^; P{13xlexAop2:IVS: TdTomato-2A-GCamP6s}attP40*	Chromosome 2 activity reporter	This work
*w^1118^; P{13xlexAop2:IVS:TdTomato-2A-GCamP6s}attP9A-VK00027*	Chromosome 3 activity reporter	This work
*w^1118^; P{Th-LexA}/CyO*	LexA driver for dopaminergic neurons	
*w^1118^ iso; P{UAS-AOX-F6}; iso*	UAS-AOX line back-crossed onto *iso^31^* background; strongly wake-promoting	Derived from Bloomington stock center #93880
*w^1118^ iso; iso; P{UAS-AOX-F24}*	UAS-AOX line back-crossed onto *iso^31^* background; moderately wake-promoting	Derived from Bloomington stock center #93881

### Transgenic guide RNA construction

The protocol of Port and colleagues was used to generate multiplex gRNA vectors for transgenesis(48). Using primers described in Supplemental Table 2, 3 PCR fragments (for each target gene) encoding tRNA-flanked exonic sgRNAs were amplified off a *pCFD5* plasmid template (Addgene #73914). Oligos were synthesized by Integrated DNA Technologies. The two largest oligos containing homology arms for Gibson assembly were PAGE-purified after synthesis to ensure correct sequence. sgRNA templates were generated using Q5 DNA Polymerase (New England Biolabs) using the following thermal cycling conditions: 98C for 30S-->32 cycles of 98C for 10, 61C (+0.5C/cycle) for 30s, 72C for 10s-->72C 2min. Gel-purified (ZymoClean Gel DNA Recovery Kit, Zymo Research) PCR amplicons were combined in a 5-fold molar excess with BbsI-linearized *pCFD5* in a Gibson assembly reaction (New England Biolabs). The *pCFD5* plasmid allows for the expression of multiple sgRNAs under the control of a single ubiquitous RNA Polymerase III promoter. Colony PCR using a GOTaq master mix (Sigma) was used to identify correctly assembled clones using the following thermal cycling parameters: 95C for 2 min--> 25 cycles of 95C for 30s, 54C (+0.5C/cycle) for 30s, 72C for 1 min-->72C for 5 min). Candidate clones were expanded in liquid culture supplemented with ampicillin (10 ug/ml) and extracted using QIAprep mini plasmid kits (Qiagen) for Sanger sequencing. See Supplemental Table 2 for primer information. Sanger sequence-validated plasmid DNA was prepared using a Midi plasmid DNA kit (Qiagen) and injected into *y M{nos-Phi*C31*} v;; P{CaryP}attP2* by Rainbow Transgenics (Camarillo, CA, USA). Individual G0 flies were crossed to *y v;;Dr/TM3,Sb* (Bloomington stock #3261) to recover germline integrants. *vermillion+* G1s were back-crossed to recover a balanced line without the *M{nos-Phi*C31*}* transgene. To test efficacy of the transgenic gRNAs, balanced lines were crossed to an *Act5C-Cas9.P, DNAlig*^*4*^ stock to induce somatic mutagenesis early and ubiquitously: *Act5C-Cas9;* U6–3:gRNA adults were never recovered, reflecting the essential role for MCI function.

### Germline mutagenesis

An *FRT40A P{neoR}, FRT2A P{mini-w+}* (hereafter *2FRT*) chromosome stock was isogenized by standard crossing methods. Females from a M{*nos*-Cas9}; *2FRT* isogenic stock were mated with males carrying multiplex gRNA transgenes on chromosome 3. The resultant *nos-Cas9/Y; 2FRT/+; gRNA/+* males were then mated to *w*^*1118*^*; Sco/CyO* females to recover * FRT40A /Sco males carrying unique mutagenized chromosomes. New recessive lethal alleles were identified by allelic non-complementation against chromosomal deficiencies uncovering *NDUFB10* or *NDUFV1.* Non-complementing lines were crossed to females from a yw; P{GMR-Hid} FRT40A l(2)CL-1; ey>FLP stock to induce mitotic recombination in the eye. Mutagenized chromosomes causing the most severe eye malformations were chosen for genomic sequencing. Eggs from * FRT40A/CyO, weeP in-crosses were collected on standard apple juice/agar plates for ~16–18h at 25°C and aged for 24h at 25°C. GFP- larvae were picked under a fluorescence stereomicroscope and homogenized in 50 ul of squish buffer (10mM Tris, 25 mM NaCl, 1 mM EDTA supplemented with 200 ug/ml Proteinase K), followed by activation of the Proteinase K under these conditions: 30 min at 37C, 2 min at 95C. See Supplemental Table 2 for primers used for PCR amplification and Sanger sequencing of *NDUFB10* and *NDUFV1* genomic regions. Taq DNA Polymerase (New England Biolabs) was used in the following thermal cycling conditions: 95C for 3 min-->35 cycles of 95C for 30s, 51C for 30s, 72C for 1:15s-->72C for 5 min. Sequences from mutants were compared to those derived from the *w*^*1118*^*; 2FRT* stock. Alignments to the reference genome (r6.65, Flybase.org) were performed using SnapGene. Mutant and the parental chromosome were back-crossed for >5 generations onto an isogenic *w*^*1118*^ genetic background.

### Mitotic clone generation

Eggs were collected at 25C for ~18h and aged to 48–66h after egg-laying. FLP expression was induced using a 38C heat shock for 30 min. Brains from females 2–4 days old adults were fixed for immunostaining.

### Survival analysis

100 mated 1–3 day old female flies, split into 5 vials of 20, were collected for each genotype. Flies were transferred to 29C for the duration of the experiment and flipped every 2–3 days onto fresh food. At each flip, the number of dead/drowned flies were counted for each vial. Flies lost during transfer were censored. The experiment was run until all or most of the knock-down flies had died. Each experiment was repeated twice to ensure reproducibility of the results. Data were analyzed by the Kaplan-Meier method in Prism.

### Generation of lexAop:TdTomato-2A-GCamP6s reporter

A PspXI-TdTomato-2A-GCamP6s-XbaI fragment was PCR amplified from Addgene plasmid #130666 and subcloned into the PspXI and XbaI sites of the linearized backbone of Addgene plasmid #111548 using standard subcloning methods. This placed the TdTomato-2A-GCamP6s coding sequencing under the control of a 13xLexAop promoter. See Supplemental Table 2 for PCR and sequencing primers used. PhiC31 integrase was used to recombine the attB site on the plasmid bakcbone at chromosomal attP dock sites on chromosome 2 (attP40) and chromosome 3 (attP9A). Germline transformants were selected by mini-w+ expression off the integrated transgene and stable lines were back-crossed onto a *w*^*1118*^ *iso*^*31*^ background. Injections were performed by Rainbow Transgenics (Camarillo, CA, USA).

### Quantitative PCR

UAS-dsRNAs were expressed using the pan-neuronal *elav*-GAL4 driver and compared to a GAL4+/dsRNA- control of the same genetic background (e.g. *elav>+ attP2)*. ~15 heads were used per genotype per biological triplicate. Total RNA was isolated from whole heads flash-frozen on dry ice and homogenized in Trizol (Invitrogen). For *NDUFV1* knock-down quantification, dissected brains from wandering third instar larvae (~25 per replicate) were used. Briefly, nucleic acids were extracted in a 5:1 mixture of Trizol: chloroform. Following centrifugation (12,000*xg* at 4C, 15 min), the upper polar phase was subjected to isopropyl alcohol precipitation (1:1 aqueous phase: isopropyl alcohol, 20 ug glycogen). Nucleic acid pellets were washed in 75% ethanol, dried, and then resuspended in 30 ul DEPC-treated ddH20. DNAse digestion was performed using Turbo^®^ DNase (ThermoFisher) using manufacturer’s protocol. Total RNA concentration was measured using a NanoDrop spectrophotometer. 1 ug of total RNA was used for first-strand cDNA synthesis (SuperScript RT II, ThermoFisher) primed with random hexamers (ThermoFisher). qPCR primers for each target cDNA were designed using NCBI Primer Blast. Oligos were synthesized by Integrated DNA Technologies and used at a concentration of 0.2uM. SYBR^®^ green PCR master mix (Applied Biosystems) was used. PCR reactions were run using the BioRad CFX Opus 96 thermal cycler. The amplification efficiency of each primer pair and control primers (see Supplementary Table 2) was calculated from the slope (efficiency= 10^−(1/slope)^ −1) of the linear regression of Ct values obtained from amplification off a 5-fold dilution series of wild-type whole-head cDNA. qPCR primers amplifying single amplicons with >95% efficiency were used in calculating relative mRNA abundance. The ΔΔCt method was used to calculate relative mRNA abundance. Data were analyzed using Excel and statistics performed using GraphPad Prism software.

### Mitochondrial Complex I assays

Mitochondrial fractions were prepared from gently homogenized flies following the protocol of Groen and Windebank(49). Mitochondria were isolated and resuspended on ice in 70 mM sucrose, 210 mM mannitol, 5 nM HEPES, 1 mM EGTA, and 0.5% bovine serum albumin, pH 7.5. A 1 ul aliquot was used to measure protein concentration using a Pierce^™^ BCA assay kit (Thermo Scientific). Absorbance of 560 nm light was measured using a plate reader. The Mitochondrial Complex I Colorimetric assay from TriBiosciences was used according to manufacturer’s protocol. MCI activity was normalized to protein amount in each sample.

### Immunostaining

Adult brains were dissected in cold 1X PBS and fixed in 4% paraformaldehyde (Electron Microcsopy Sciences) for 20 min. Tissue was permeabilized in 0.3% Triton X-100 in 1X PBS (PBST) for 20 min. All steps performed at room temperature with nutating. Blocking was performed in 5% goat serum in PBST for >1h at room temperature or overnight at 4C. Primary antibodies were diluted in PBST with 5% goat serum and incubated with nutating at 4C overnight. Secondary antibodies were diluted 1000-fold in PBST and incubated with brains for 2h at room temperature or overnight at 4C. Between antibody incubations, brains were washed in PBST at room temperature with nutating for 20 min. Before mounting, PBST was replacted with 1X PBS and the brains were mounted on slides with a 2:1 mixture of SloFade mounting media: 1X PBS. Imaging spacers were placed between the slide and coverslip. Primary antibodies used were: Chicken anti-GFP (Invitrogen) at 1:1000, Rabbit anti-mCherry at 1:100 (GeneTex), rabbit anti-Tyrosine Hydroxylase at 1:100 (BD Biosciences), and nc82 at 1:100 (Developmental Studies Hybridoma Bank). Secondary antibodies (Invitrogen) used were: Goat anti-Chicken Alexa 488, Donkey anti-Rabbit Alexa 555, and Donkey anti-mouse Alexa 647.

### Microscopy

Imaging was performed on a Leica SP6 laser scanning confocal microscope (Leica Microsystems) equipped with 488 nm, 514 nm, and 613 nm lasers. 512 × 512 images z-stacks (1.5–2um step sizes) were collected by bidirectional linear scanning with 2x line averaging. For fixed and stained samples, scanning was performed at 400Hz. Where quantitation of intensity was performed across conditions, laser power and gain were kept constant across conditions. For qualitative imaging, power and gain were adjusted to bring pixel intensity just below saturation. For *ex vivo* GCamP imaging, brains were rapidly dissected during ZT1–3 and mounted in a drop of cold Artificial Hemolymph [***] caudal side up on poly-l-lysine slides with imaging spacers and a coverslip. Laser scanning was performed at 8000Hz using resonant scanning using 488 nm and 514 nm lasers. Only the PPL1 DAN cell bodies closest to the objective were imaged to minimize light scattering. Linear bidirectional scanning with 2x averaging and a 72 ns pixel dwell time was used throughout all experiments. Laser power and gain were kept constant during imaging sessions. Single optical slices containing the majority of cell body signal were used for acquiring images every 30s for ~2.5–5 min to assess the stability of GCamP fluorescence. GCamP6S fluorescence never varied across time-lapse imaging, but the same frame (t=3) was used for all image quantitation. A 3×3 smoothing filter in ImageJ was applied to the EGFP and TdTomato channels before calculating the background-subtracted mean intensity of each cell body. The ratio of mean GCamP/TdTomato intensities was calculated for each cell body. All mean intensity measurements were made using ImageJ.

## Supplementary Material

Supplement 1

## Figures and Tables

**Table 1:** Summary of phenotypes from pan-neuronal MCI manipulations

	*elav*-GAL4^c155^>		
	UAS-dsRNA		UAS-Cas9 U6:3-sgRNAs
Target	Phenotype	mRNA levels (% WT +/− SD)	Phenotype
*NDUFV1*	Pupal lethal	28.8 +/− 0.04% (p=0.0004)	n/a
*NDUFB 10*	Adult viable	43.8 +/−10% (p=0.014)	pupal lethal
*NDUFS2*	Adult viable	36.5 +/−1.7% (p=0.003)	
*NDUFAF4*	Adult viable	45.6 +/−4.5% (p=0.0002)	n/a

Hypoactive

Short-sleeping

The *elav-GAL4* pan-neuronal GAL4 driver was used to manipulate MCI subunit function either using RNAi or somatic CRISPR-mediated mutagenesis using a UAS-Cas9 transgene. P values computed using Welch’s t tests.

## Data Availability

All data needed to evaluate the conclusions in the paper are present in the paper and/or the Supplementary Materials.
