# Supplementary material for: Reverse electron transfer at mitochondrial complex I restrains dopaminergic neuron activity to promote early-life sleep in Drosophila
Source: bioRxiv. 2026 Feb 23:2026.02.22.707308. Preprint. [Version 1] doi: 10.64898/2026.02.22.707308 (PMC13160131; doi:10.64898/2026.02.22.707308)

bioRxiv preprint doi: https://doi.org/10.64898/2026.02.22.707308; this version posted February 23, 2026. The copyright holder for this preprint (which was not certified by peer review) is the author/funder, who has granted bioRxiv a license to display the preprint in perpetuity. It is made available under aCC-BY 4.0 International license.

**Supplemental Figure 1: Differential expression analysis of juvenile and mature dopaminergic neurons**

(A) GO analysis of differentially expressed genes chosen for RNAi screening. Genes preferentially expressed in juvenile DANs were more likely to encode components of metabolic pathways (examples highlighted in red). (B) KEGG pathway analysis of the same gene set revealed a similar enrichment of metabolic pathways with the highest enrichment mapping to components of the oxidative phosphorylation pathway. (C) Volcano plot of differential expression showing genes of interest (GOIs, cyan) chosen for the transgenic RNAi screen.

**Supplemental Figure 2: Additional sleep metrics for *Th*>MCI RNAi flies**

(A) Night sleep was unaffected in *Th>NDUFB10* IR and *Th>NDUFS2* IR short-sleeping flies. (B) Night sleep was unaffected in *Th>NDUFV1* IR long-sleeping flies. (C) Day sleep duration and waking locomotor activity plotted for each *Th>NDUFV1* IR fly (red dots). The duration of day sleep was significantly correlated with reduced locomotion (Spearman r=-0.45, p=0.0331), indicating variability in sleep can be explained by changes in locomotor behavior. (D-G) Multibeam sleep data for mature adult

bioRxiv preprint doi: https://doi.org/10.64898/2026.02.22.707308; this version posted February 23, 2026. The copyright holder for this preprint (which was not certified by peer review) is the author/funder, who has granted bioRxiv a license to display the preprint in perpetuity. It is made available under aCC-BY 4.0 International license.

*Th>NDUFAF4* IR flies. Daytime sleep loss was variable in these flies, though many slept far less than controls. (H-J) Cumulative frequency plots of sleep bout duration for short-sleeping Th>MCI RNAi lines.  Knock-down adult flies did not show a consistent difference in sleep bout length relative to both control (ns, not significant; *** $p<0.001$ **** $p<0.0001$). P values computed using either Kruskal-Wallis tests with Dunn's multiple-testing comparisons or Welch ANOVA with Dunnett's T3 multiple-testing comparisons.

bioRxiv preprint doi: https://doi.org/10.64898/2026.02.22.707308; this version posted February 23, 2026. The copyright holder for this preprint (which was not certified by peer review) is the author/funder, who has granted bioRxiv a license to display the preprint in perpetuity. It is made available under aCC-BY 4.0 International license.

**Supplemental Figure 3: Germline mutants for *NDUFV1* and *NDUFB10* and their phenotypes**

(A,B) Position and Sanger chromatograms of germline mutations induced by CRISPR/Cas9. Wild-type sequence derived from isogenized *FRT40A, FRT2A (2FRT*) chromosome. Position of single guide RNAs used shown in red (PAM sequences in black boxes). Shaded red indicates sgRNA that did not induce indels. (C) Mitotic recombination was induced in eye precursor cells. Mutants in *NDUFB10* and *NDUFV1* caused small, glossy eyes similar to those reported for other OXPHOS pathway genes. *NDUFB10*$^{ts}$ clones were overtly normal at 25°C but abnormal at 29°C. (D) *NDUFB10*$^{ts}$ can rescue the lethality of *NDUFB10*$^{33}$ at 22°C and 18°C but not higher temperatures. (E) ClustalOmega alignment of a conserved disulfide bridge in NDUFB10. Both mutant alleles affect a conserved intramolecular disulfide bridge.  The NDUFB10$^{ts}$ allele deletes 4 amino acids predicted by AlphaFold (*) to stabilize the disulfide bonded alpha helices. (F) Complex I assays  in mitochondria isoalted from *2FRT*  wild-type flies and *NDUFB10*$^{ts}$ mutants reared at 22°C during development and shifted to 29°C  for 1 week to inactivate the NDUFB10$^{ts}$ allele. Mitochondria from the mutants exhibited reduced MCI activity. P value computed using a MAnn-Whitney test.  (G) Axon terminals of GFP-labeled wild-type (*2FRT*) and *NDUFB10*$^{33}$ PPL1 clones projecting to the dorsal fan-shaped body (arrowheads. Neuropil (magenta) labelled by nc82 mAb. (H) The frequency (clone #/brain) and overall distribution of DAN MARCM clones recovered. The *NDUFB10*$^{33}$ mutation did not impair clone survival. P value were computed using a Mann-Whitney t-test.  (I) The axon arbor area of wild-type and *NDUFB10*$^{33}$ clones

bioRxiv preprint doi: https://doi.org/10.64898/2026.02.22.707308; this version posted February 23, 2026. The copyright holder for this preprint (which was not certified by peer review) is the author/funder, who has granted bioRxiv a license to display the preprint in perpetuity. It is made available under aCC-BY 4.0 International license.

synapsing onto the dorsal fan-shaped body. The *NDUFB10*[33] mutation did not impair axon pathfinding and innervation of DANs. P value was computed using Welch's t-test.

**Supplemental Figure 4: MCI activity in DANs is required for the morning and evening activity peaks.**

(A,B) Raw activity counts binned into 3h intervals for strong MCI LOF (*Th>NDUFV1* IR, red) and partial MCI LOF (*Th>NDUFB10* IR, blue) flies. Strong MCI, but not partial MCI, LOF severely depressed morning (ZT0-3) and evening (ZT9-12) activity peaks. P values computed using Kruskal-Wallis tests with Dunn's multiple testing comparisons. (C) Wild-type PPL1 DANs expressing TdTomato-2A-GCamP6s and imaged *ex vivo*. The steady-state GCamP/TdTomato ratio was higher in the morning (ZT0-3) than the afternoon (ZT4-8) during the afternoon siesta. P value computed by a Mann-Whitney t-test. (D-F) Confirmation of Cas9 mutagenic activity in DANs on the *EGFP* coding sequence. Reduced or loss of EGFP (green) signal indicates Cas9-mediated mutagenesis. Some DAN clusters (PPM1/2 and PPL1) showed highly uniform Cas9 activity, while other clusters (PPM3 and PPL2A) had more heterogeneous activity. Mean EGFP intensity in DAN cell bodies was measured in the absence (green) or presence (grey) of Cas9. Data are normalized to the median of the control. P values computed using Mann-Whitney tests. (G,H) Quantification of evening waking activity for MCI RNAi (top) and MCI somatic knock-out (bottom) flies. Red corresponds to hypoactive conditions, blue to short-sleepers. P values computed using Kruskal-Wallis tests with Dunn's multiple-testing comparisons and Welch ANOVAs with Dunnett's T3 multiple-testing comparisons.

bioRxiv preprint doi: https://doi.org/10.64898/2026.02.22.707308; this version posted February 23, 2026. The copyright holder for this preprint (which was not certified by peer review) is the author/funder, who has granted bioRxiv a license to display the preprint in perpetuity. It is made available under aCC-BY 4.0 International license.

**Supplemental Figure 5: Knock-down of MCI in *Th*+ cells shortens lifespan and causes age-dependent DAN loss**

(A)Survival analysis of Th>*NDUFV1* IR (red) and Th>*NDUFB10* IR (blue) flies, and genetic controls, aged at 29C. Strong MCI loss-of-function (red) caused a more severe truncation of lifespan (Median survival=15 +/-1 days, P<0.0001 Mantel-Cox test) than partial MCI loss-of-function (blue, Median survival=20 +/- 2 days, P<0.0001 Mantel-Cox test) . (B) Quantification of DANs labelled by anti-Tyrosine Hydroxylase immunostaining in aged (~21 days at 29C) *Th*>*NDUFB10* IR and *Th*>+ control flies showing reduced PPL1 DAN counts in knock-down flies. P value computed using Mann-Whitney test.

bioRxiv preprint doi: https://doi.org/10.64898/2026.02.22.707308; this version posted February 23, 2026. The copyright holder for this preprint (which was not certified by peer review) is the author/funder, who has granted bioRxiv a license to display the preprint in perpetuity. It is made available under aCC-BY 4.0 International license.

**Supplemental Figure 6: Disinhibition of DA signaling by withdrawal of the light cue modifies wild-type sleep, but not MCI-deficient, flies.**

A) Sleep during ZT0-12 (lights on) or CT0-12 (no lights on, shaded) for *Th>NDUFB10 IR* mature flies (blue) and controls (grey). Sleep loss in the knock-down flies persisted in constant darkness. Control flies showed a pronounced sleep loss compared to the previous day with lights on. This phenotype was abrogated in *Th>NDUFB10* IR flies. (B) Change in day sleep [Day sleep(DD)-Day sleep (LD)] plotted for each fly in the experiment in B. Values < 0 (dotted line) indicate loss of sleep. *NDUFB10* knock-down abrogated sleep loss due to withdrawal of the light cue. (C )Change in P(wake) [P(wake)(DD)-P(wake)(LD)] plotted for each fly in panel A. Sleep loss upon withdrawal of the light cue was driven by an increase in P(wake)--an effect attenuated *NDUFB10* knock-down. (D) Change in P(doze) [P(doze)(DD)-P(doze)(LD)] plotted for each fly in panel B. Withdrawal of the light cue caused a slight but significant decrease in P(doze) that was not affected by *NDUFS2* knock-down. (E,F) Change in night sleep for

bioRxiv preprint doi: https://doi.org/10.64898/2026.02.22.707308; this version posted February 23, 2026. The copyright holder for this preprint (which was not certified by peer review) is the author/funder, who has granted bioRxiv a license to display the preprint in perpetuity. It is made available under aCC-BY 4.0 International license.

*Th>NDUFB10* IR mature flies (blue) and genetic controls (grey) (mean and interquartile range shown). P values calculated by Wilcoxon signed rank tests (* $P<0.05$, ** $P<0.01$, **** $p<0.0001$ compared to $\Delta=0$). Mean and standard deviation plotted for each graph.

bioRxiv preprint doi: https://doi.org/10.64898/2026.02.22.707308; this version posted February 23, 2026. The copyright holder for this preprint (which was not certified by peer review) is the author/funder, who has granted bioRxiv a license to display the preprint in perpetuity. It is made available under aCC-BY 4.0 International license.

**Supplemental Figure 7: Additional juvenile sleep metrics for partial MCI loss-of-function.**

(A-D) Juvenile sleep phenotypes for *Th>NDUFAF4* IR juvenile flies and controls. Knock-down flies exhibited significant daytime sleep loss (A) driven by reduced bout duration (C, D, **** p<0.0001 Kruskal-Wallis with Dunn's multiple comparisons test). (E-G) Cumulative frequency plots for durations of nighttime sleep bouts of partial MCI knock-down flies. Juvenile flies for all three knock-down conditions showed significantly reduced bout length during the night (** p<0.01, *** p<0.001, **** p<0.0001 Kruskal-Wallis with Dunn's multiple testing comparisons ). (H) P(wake) (i) and P(doze) (ii) measurements (average and SEM) for 30 min windows across ZT0-24 for juvenile *Th>NDUFS2* IR juvenile flies (blue) and controls (grey). Knock-down flies showed significantly elevated P(wake) across ZT0-12, indicating sleep loss during this period was driven by reduced sleep depth. P(doze) was only visibly reduced compared to both controls during ZT6-12. (I) P(wake) (i) and P(doze) (ii) measurements (average and SEM) for 30 min windows across ZT0-24 for juvenile *Th>NDUFAF4* IR juvenile flies (blue) and controls (grey). Juvenile *Th>NDUFAF4* IR sleep loss was driven by elevated

bioRxiv preprint doi: https://doi.org/10.64898/2026.02.22.707308; this version posted February 23, 2026. The copyright holder for this preprint (which was not certified by peer review) is the author/funder, who has granted bioRxiv a license to display the preprint in perpetuity. It is made available under aCC-BY 4.0 International license.

P(wake). (J) Time to first sleep bout after lights-off (ZT12) for juvenile *Th>NDUFS2* IR juvenile flies and genetic controls, showing no difference across genotypes. (K) Waking locomotor activity during morning (ZT0-3) for *Th>NDUFS2* IR juvenile flies and genetic controls. Despite increased sleep latency during this period (see Fig 4), knock-down flies did not exhibit elevated locomotor activity, indicating prolonged sleep latency is not a secondary consequence of hyperactivity. P values computed using either Kruskal-Wallis tests with Dunn's multiple-testing comparisons or Welch ANOVA with Dunnett's T3 multiple-testing comparisons.

bioRxiv preprint doi: https://doi.org/10.64898/2026.02.22.707308; this version posted February 23, 2026. The copyright holder for this preprint (which was not certified by peer review) is the author/funder, who has granted bioRxiv a license to display the preprint in perpetuity. It is made available under aCC-BY 4.0 International license.

**Supplemental Figure 8: Depletion of dopaminergic CoQH2 by multiple, independent means caused sleep loss.**

(A) Quiescence per 30 min windows (mean with 95% confidence interval shaded) for *Th>ATPsynCF6* IR mature adult flies (red), expressing a dsRNA against an essential structural component of the F(1)-F(0) ATPase, and genetic controls (grey). The dramatic increase in daytime quiescence reflects severely reduced locomotion (B, $p<0.0001$ Welch's ANOVA with Dunnet's T3 multiple comparisons test). (C) Quantitative PCR of whole-head cDNA from *elav>sdhAF4* IR flies (blue) and the landing site control (grey). The two independent short-sleeping RNAi lines targeting *sdhAF4* comparably reduced *sdhAF4* mRNA levels. P value computed using Welch ANOVA and Dunnett's T3 multiple

bioRxiv preprint doi: https://doi.org/10.64898/2026.02.22.707308; this version posted February 23, 2026. The copyright holder for this preprint (which was not certified by peer review) is the author/funder, who has granted bioRxiv a license to display the preprint in perpetuity. It is made available under aCC-BY 4.0 International license.

comparisons test. (D) Juvenile sleep duration during ZT0-12 for *Th>sdhAF4* IR #2 (blue) and controls (grey) assayed using the multibeam activity monitoring system, confirming sleep loss phenotype reported in primary screen. (E) P(wake) plotted in 30 min windows (average with SEM) for juvenile *Th>sdhAF4* IR #2 (blue) and controls (grey) showing a persistently elevated P(wake) during ZT0-12, indicative of increased transitions from inactive to active—a phenotype is identical to partial MCI inhibition. (F) Sleep per 30 min windows (meanwith 95% confidence interval shaded) of *Th>AOX-F24* mature adult flies (blue) expressing an independent P element insertion of UAS-*AOX* and genetic controls (grey). (G) Day and night sleep duration for mature adult *Th>AOX-F24* flies (blue). Compared to the insertion used in Fig 5, UAS-*AOX-F24* flies showed comparable sleep loss during ZT0-12 but no effect on sleep during ZT12-24. P values computed using a Kruskal-Wallis test with Dunn's multiple testing comparisons. (H, I) Sleep bout metrics for *Th>AOX-F24.* Sleep loss was driven by both reduced bout number and bout length (**** p<0.0001, Kruskal-Wallis test with Dunn's multiple testing comparisons). (J, K) P(doze) and P(wake) averaged across ZT0-12 for *Th>AOX-F24* flies (blue) and genetic controls (grey). As with UAS-AOX transgene used in Fig 5, *Th>AOX-F24* flies showed a substantial increase in P(wake), and a modest decrease in P(doze), during ZT0-12. P values computed using Kruskal-Wallis tests with Dunn's multiple-testing comparisons.

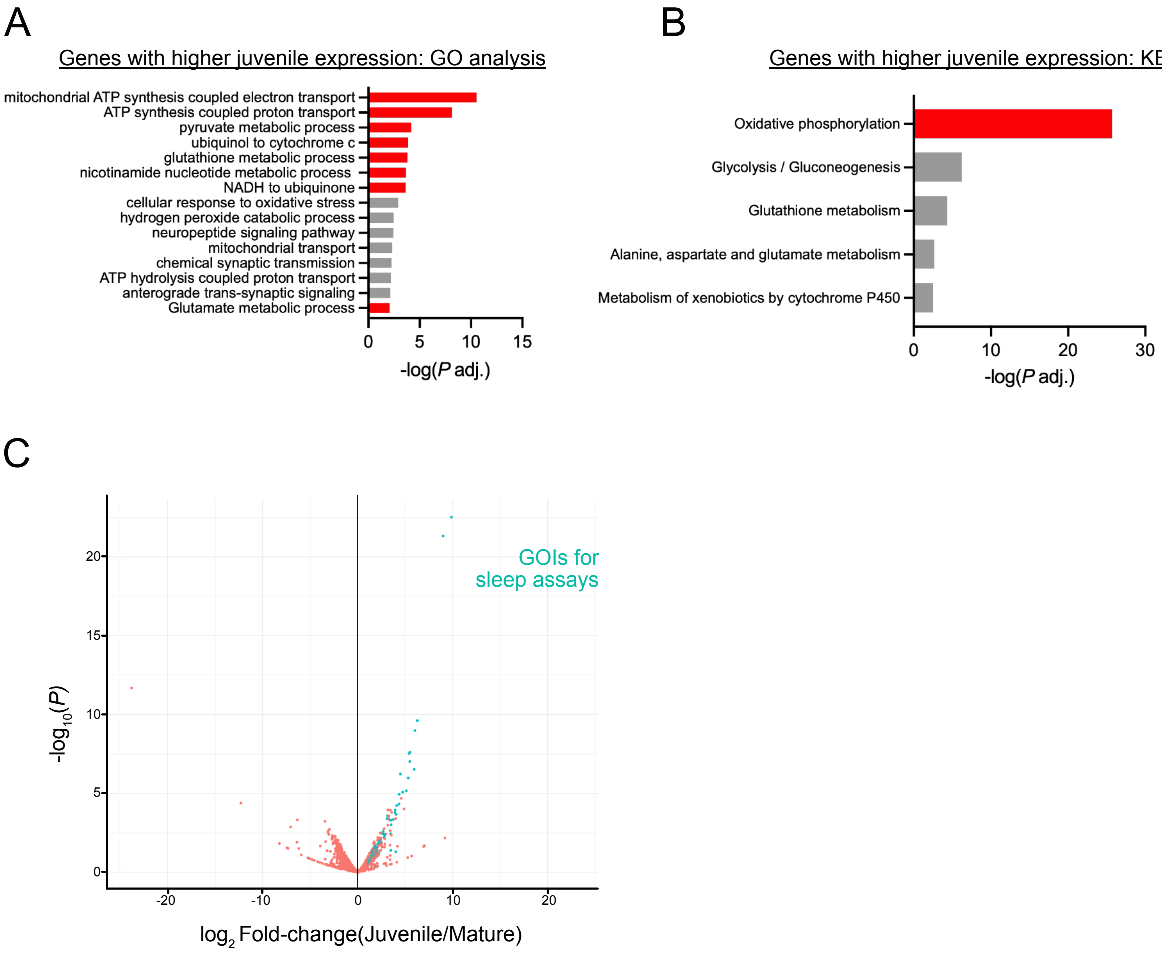


*Supplemental Figure 1*

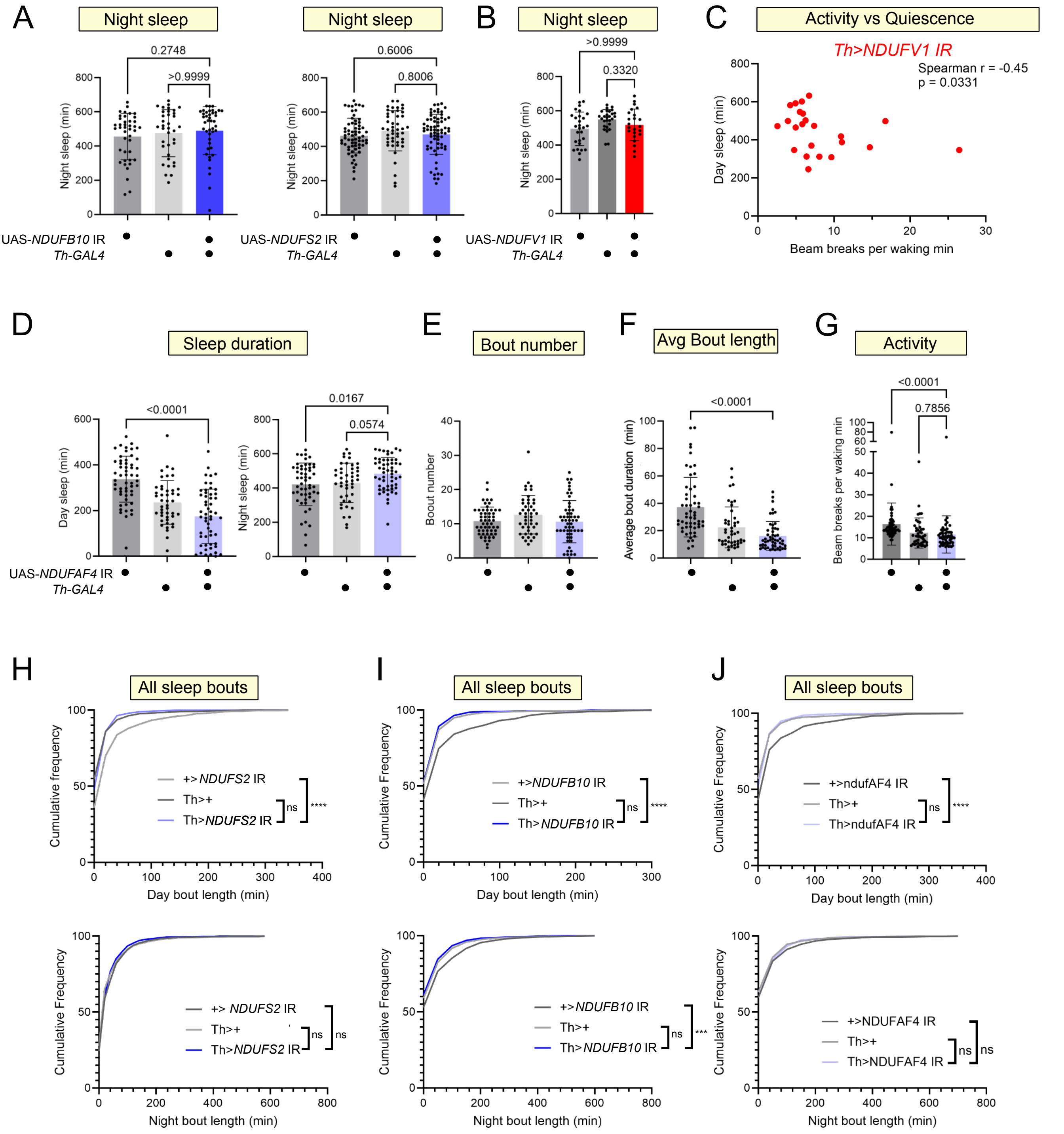


*Supplemental Figure 2*

bioRxiv preprint doi: https://doi.org/10.64898/2026.02.22.707308; this version posted February 23, 2026. The copyright holder for this preprint (which was not certified by peer review) is the author/funder, who has granted bioRxiv a license to display the preprint in perpetuity. It is made available under aCC-BY 4.0 International license.

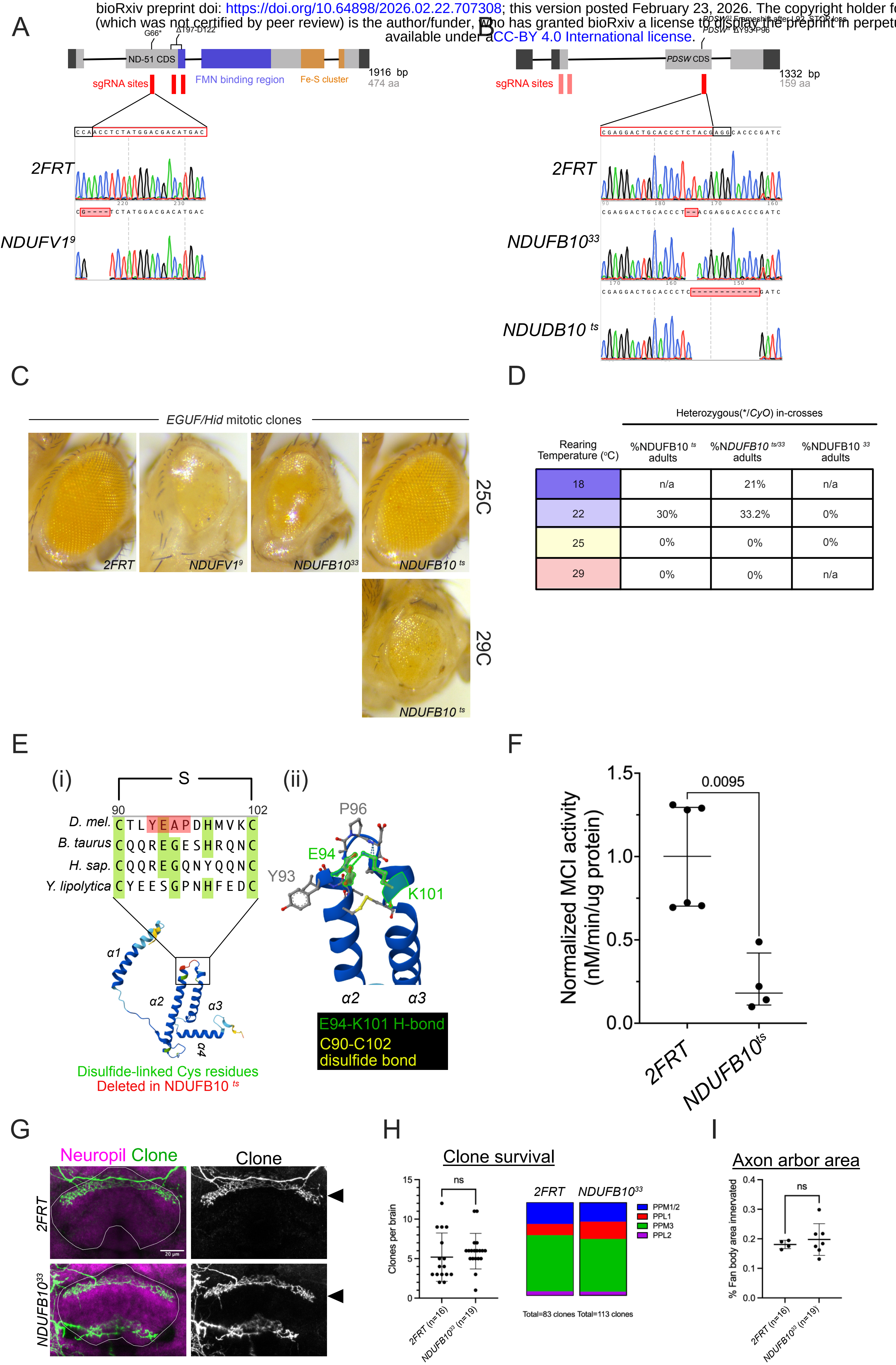


Supplemental Figure 3

bioRxiv preprint doi: https://doi.org/10.64898/2026.02.22.707308; this version posted February 23, 2026. The copyright holder fo
(which was not certified by peer review) is the author/funder, who has granted bioRxiv a license to display the preprint in perpetu
available under aCC-BY 4.0 International license.

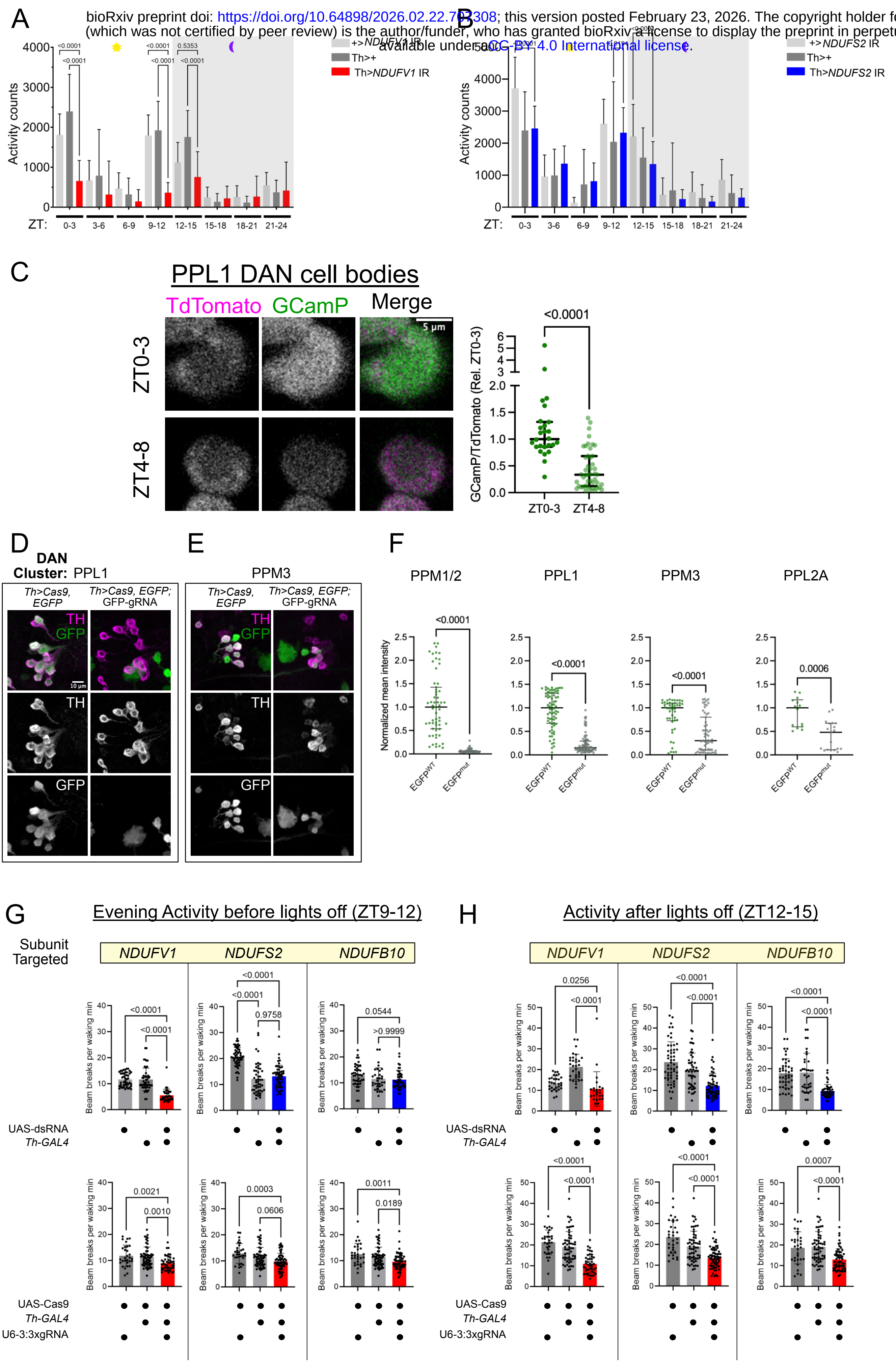


Supplemental Figure 4

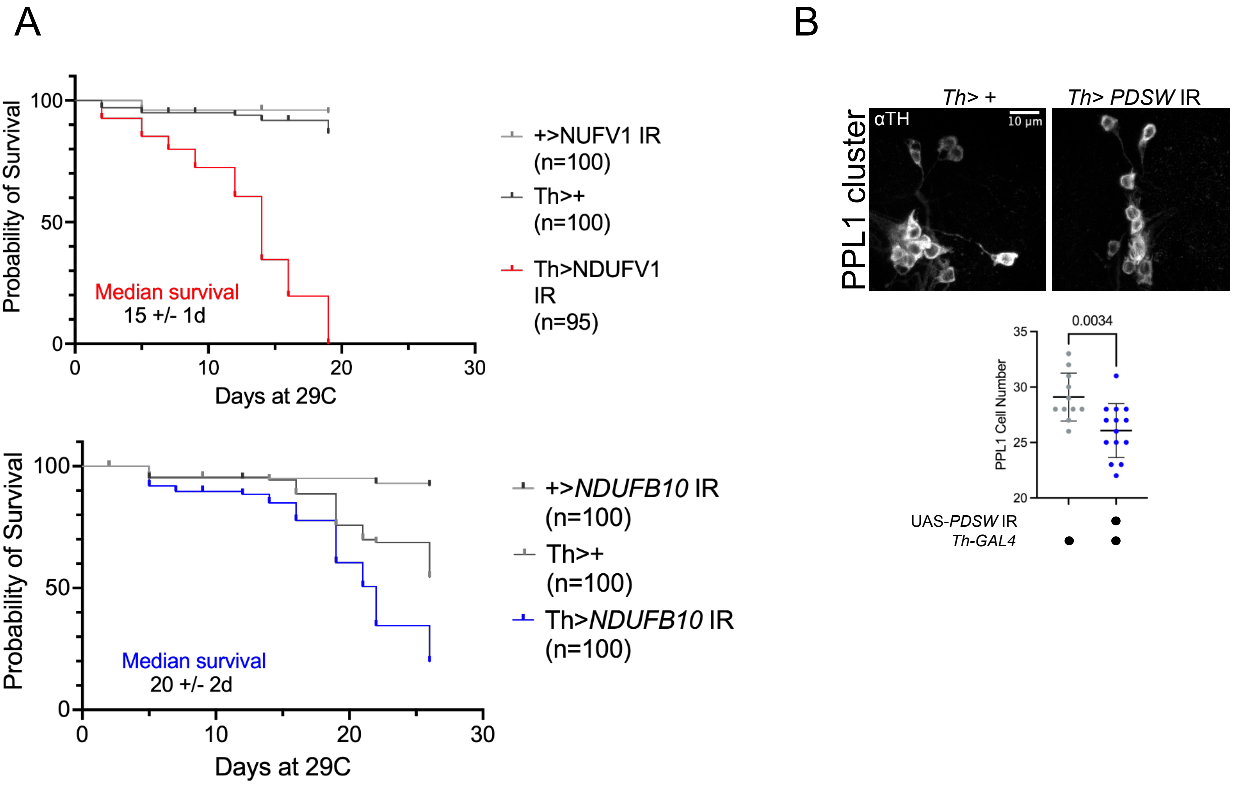


*Supplemental Figure 5*

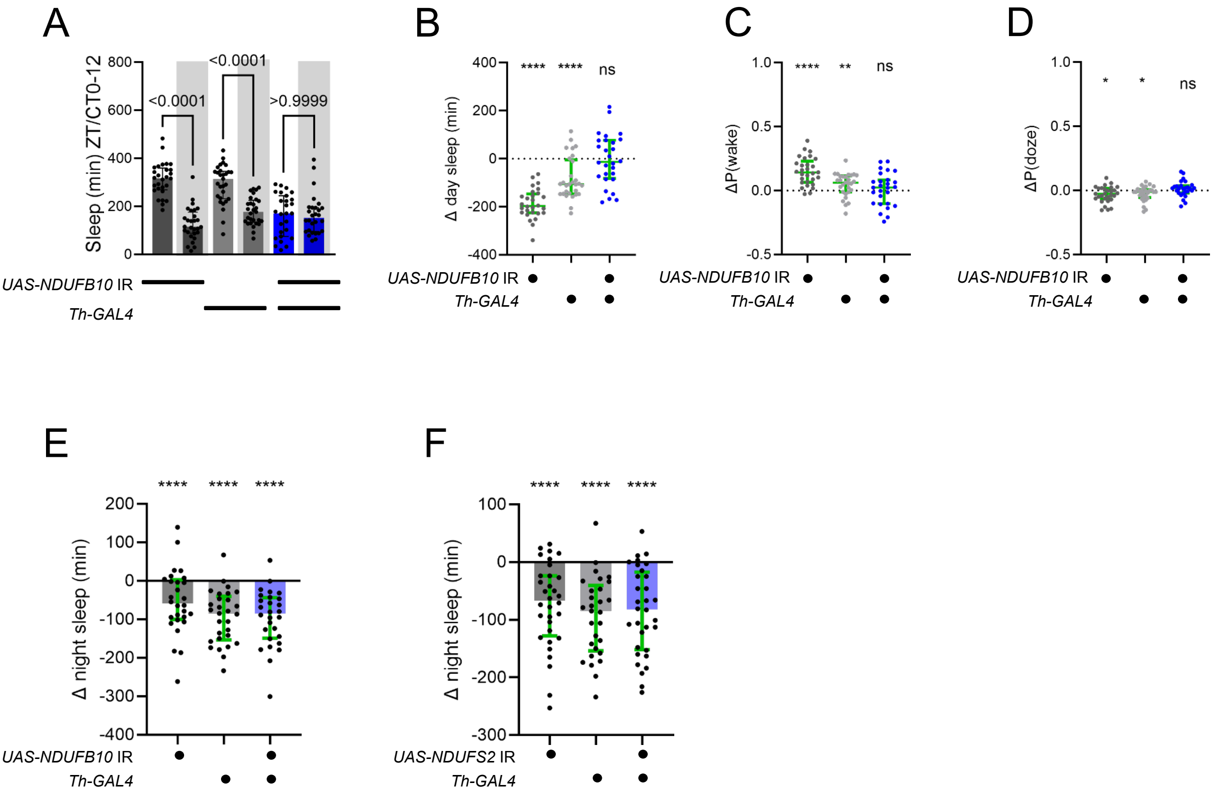


*Supplemental Figure 6*

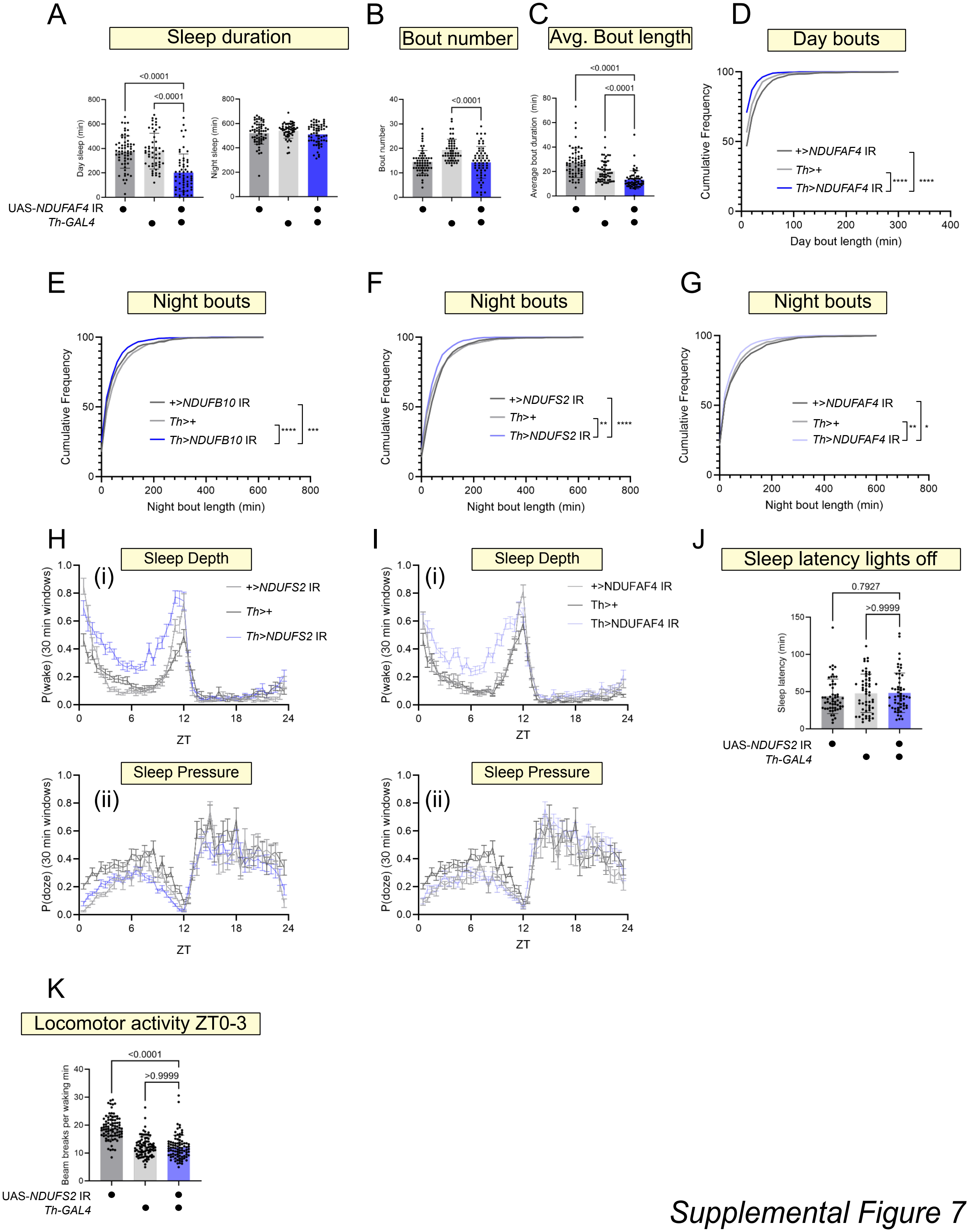


*Supplemental Figure 7*

# Supplemental Figure 8

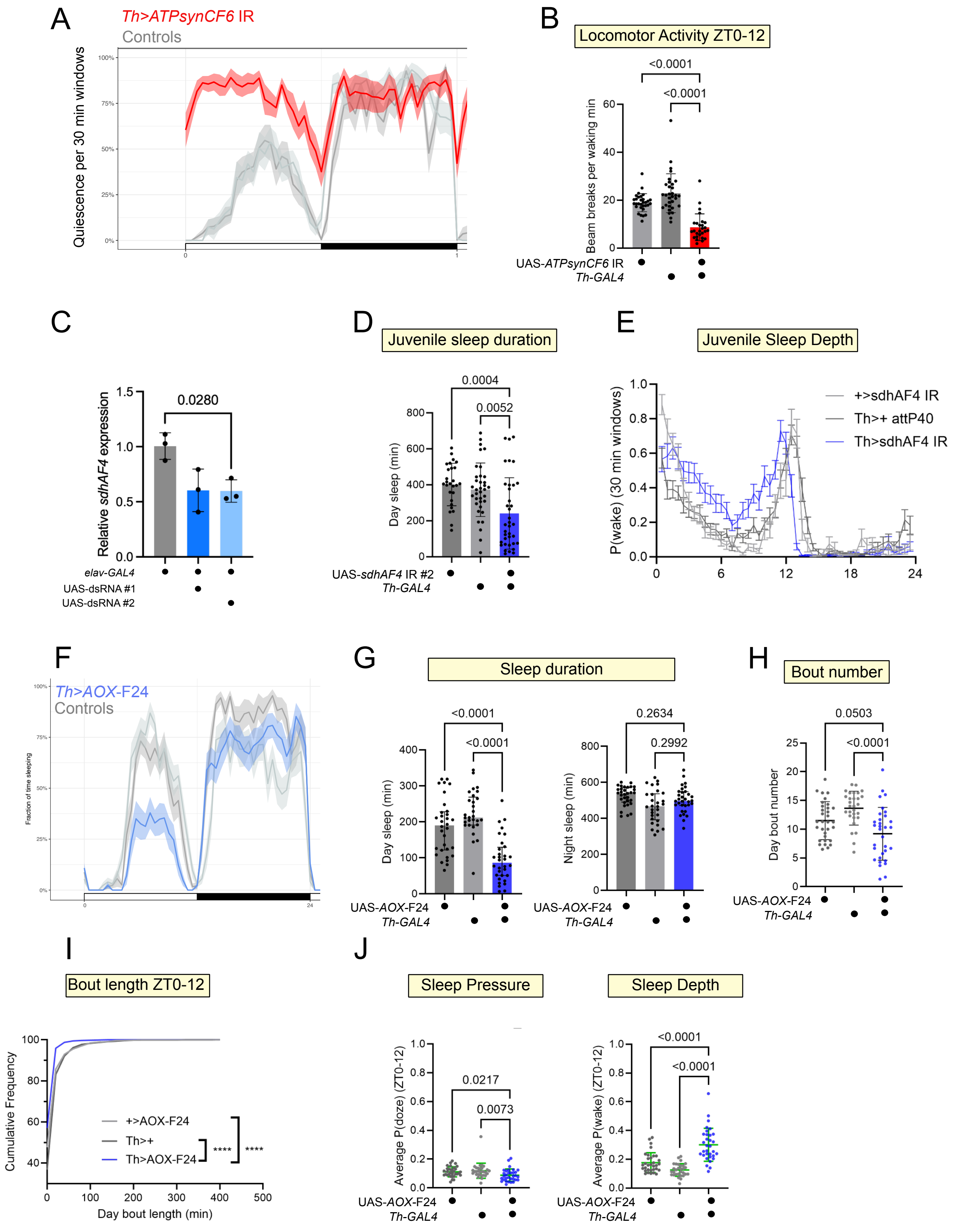

Supplement: Supplement 1 [file NIHPP2026.02.22.707308v1-supplement-1.pdf]
